# Taming microglia: the promise of engineered microglia in treating neurological diseases

**DOI:** 10.1186/s12974-024-03015-9

**Published:** 2024-01-11

**Authors:** Echo Yongqi Luo, Rio Ryohichi Sugimura

**Affiliations:** 1 School of Biological Sciences, Faculty of Science, The University of Hong Kong, Pokfulam, Hong Kong; 2 School of Biomedical Sciences, Li Ka Shing Faculty of Medicine, The University of Hong Kong, Pokfulam, Hong Kong

**Keywords:** Microglia, Microglial genetic re-engineering, Microglial therapeutic modulation, Neuroinflammation, Neurodegenerative diseases, Glioblastoma, Multiple sclerosis

## Abstract

Microglia, the CNS-resident immune cells, are implicated in many neurological diseases. Nearly one in six of the world’s population suffers from neurological disorders, encompassing neurodegenerative and neuroautoimmune diseases, most with dysregulated neuroinflammation involved. Activated microglia become phagocytotic and secret various immune molecules, which are mediators of the brain immune microenvironment. Given their ability to penetrate through the blood–brain barrier in the neuroinflammatory context and their close interaction with neurons and other glial cells, microglia are potential therapeutic delivery vehicles and modulators of neuronal activity. Re-engineering microglia to treat neurological diseases is, thus, increasingly gaining attention. By altering gene expression, re-programmed microglia can be utilized to deliver therapeutics to targeted sites and control neuroinflammation in various neuroinflammatory diseases. This review addresses the current development in microglial engineering, including genetic targeting and therapeutic modulation. Furthermore, we discuss limitations to the genetic engineering techniques and models used to test the functionality of re-engineered microglia, including cell culture and animal models. Finally, we will discuss future directions for the application of engineered microglia in treating neurological diseases.

**Graphical Abstract:**

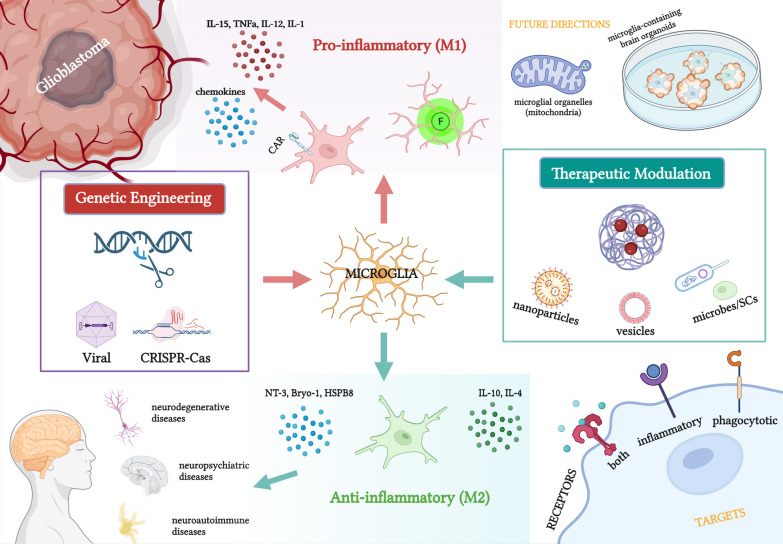

## Background

### Origin and destination of microglia

Microglia are brain-resident macrophages involved in neural development, immune responses, and cognitive functions. During early embryogenesis, microglial cell progenitors arise from the yolk sac and migrate into the brain, where they differentiate into the microglia [1]. This process is mediated/regulated by a set of transcription and growth factors. Microglia-specific transcription factor PU.1 is essential for microglia development and regulation of gene expression [2], and colony-stimulating factor-1 (CSF-1) and interleukin-34 (IL-34) bind to CSF1 receptors to regulate microglial survival, proliferation, and differentiation [3]. Microglia are localised across all brain regions and exhibit transcriptional heterogeneity, as revealed by numerous single-cell studies [4–6]. In recent years, microglia have attracted growing attention due to their active participation in different neuropathological contexts.

### Physiological roles of microglia

The understanding of microglia’s functions is still evolving. Primarily underestimated, they were merely considered structural supporters (“glia” means “glue” in Greek) and debris-engulfing phagocytes for the central nervous system (CNS). Characteristically consisting of a soma projecting elongated ramified processes, they alter phenotypes (ramified or amoeboid/reactive) to tune motility and functions [7].

The discovery of major histocompatibility complex (MHC)-II on microglia unravelled their role in CNS immunity as the major CNS-resident immune cells [8]. In response to axonal injuries, activated microglia remove myelin debris, followed by necroptosis of pro-inflammatory microglia and repopulation of anti-inflammatory microglia, to promote remyelination by oligodendrocytes [9]. Being professional phagocytes, they phagocytose misfolded proteins, pathogens, and apoptotic cells, usually following rapid proinflammatory responses. This maintains the homeostatic state of the brain by preventing autoimmune neuroinflammation and minimising unnecessary damage to the brain [10]. For example, the phagocytosis of apoptotic cytotoxic T lymphocytes helps terminate the inflammatory response [11].

Besides homeostasis maintenance mediated by immune responses, they are involved in brain development and cognitive functions. Synaptic pruning, enabled by microglial phagocytosis, stresses the intimate neuron–microglia interaction during brain development and in mature CNS, in the form of a “quad-partite” synapse [12, 13]. Amazingly, they are involved in higher cognitive functions, including learning and memory: microglia can eliminate synapses, in a complement-dependent way, to mediate forgetting [14]; dynamically surveying and modulating plastic plasticity, they might reshape neuronal circuits related to different brain functions [15]. With increasing attention on microglia, more discoveries on their functions are ongoing.

### Dysfunctional microglia in the diseased brain

Microglia out of homeostasis will lead to a wide range of neurological diseases. Constitutively activated microglia secrete pro-inflammatory cytokines (e.g., IL-1 $\beta$, IL-6, TNF-$a$) that trigger infiltration of peripheral immune cells, e.g., T cells and monocytes, that exacerbate neuronal cell death [16–19]. Chronic neuroinflammation creates an ROS-laden microenvironment that discourages regeneration, and it has been implicated in many neurodegenerative diseases [20]. For example, Alzheimer’s disease (AD) is characterised by the neurotoxic accumulation of intracellular neurofibrillary tau-tangles and extracellular A-beta plaques. These misfolded protein aggregates activate microglial immune responses by their pattern recognition receptors, e.g., toll-like receptors coupled with coreceptors CD14 and CD36, and TREM2, triggering receptors expressed on myeloid cells [21]. Considered drivers of tau pathology, reactive microglia help spread abnormal tau to more brain regions, resulting in progressive neurodegeneration contributing to memory loss in AD [22]. Besides this indirect damage to neurons, over-reactive microglia actively demyelinate neuronal axons and inhibit the functions of myelin-producing oligodendrocytes in multiple sclerosis [23]. Not only genetic factors, external stressors, such as unhealthy habits and overconsumption of drugs/alcohol, can lead to the over-activation of microglia in the brain, implicated in a wide range of neuropsychiatric diseases [24, 25]. To conclude, over-reactive microglia can cause a series of undesirable consequences, and so do under-reactive microglia.

Underperforming microglia facilitate the progression of glioblastoma (GB), a malignant brain tumour with an immunosuppressive tumour microenvironment [26]. GB-associated microglia (GAM) demonstrate a pro-tumour phenotype, following the uptake of extracellular vesicles released by GB cells [27]. GAMs are less capable of detecting tumour cells, and they release anti-inflammatory mediators and transforming growth factors that support tumour growth and invasion, which might severely hamper brain functions [28, 29]. Therefore, the homeostasis of microglia activation is important for health and diseases in the brain.

Maintaining the homeostasis of microglia is key to brain health. Restoring the disrupted homeostasis in the microglia might treat neurological diseases with dysfunctional microglia underlying. This article reviews how engineered microglia are being utilised to treat neurological conditions. Focusing on the manipulation, genetic engineering, and the in vivo and in vitro applications of microglia, we summarise current developments in this field and demonstrate the tantalising prospect of engineered microglia as treatments for neurological diseases.

## Re-engineering microglia to treat neurological diseases

**Fig. 1:**
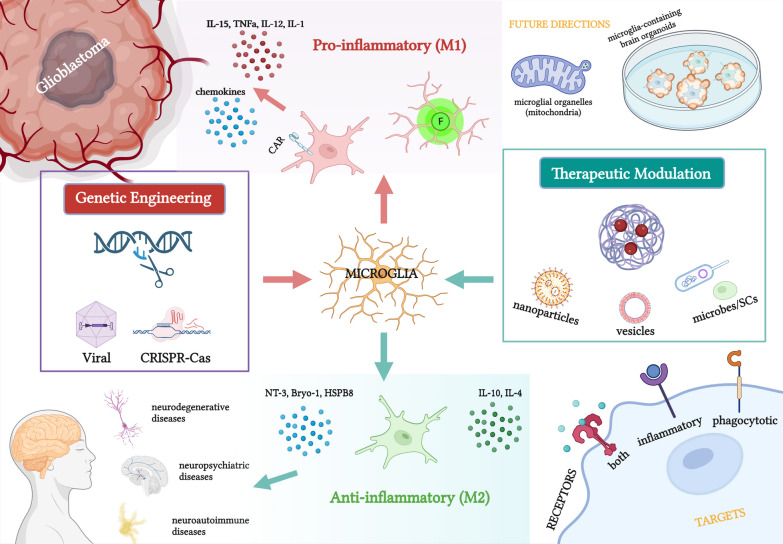
Graphical abstract of this review. Re-engineered microglia can be created by genetic engineering (viral- or CRISPR–Cas-mediated) or therapeutic modulation (nanoparticles, extracellular vesicles, or reprogrammed microbes/stem cells). Re-programmed microglia serve as therapeutic carriers and microenvironment modulators to treat a wide range of neurological diseases. This figure was created with https://www.biorender.com

The application of genetically re-engineered microglia in different neurological diseases ranges from glioblastoma/gliomas to neurodegenerative diseases, and neuropsychiatric diseases to neuroautoimmune diseases. This can be achieved by the insertion, deletion, or editing of target genes. Inserting therapeutic genes (e.g., specific cytokines and neurotrophin-3) via viral transduction or nucleic acid-loaded nanogel can impart microglia with therapeutic effects, and expression of chimeric antigen receptors on the microglial surface can increase targeting efficiency. Some are introduced with green fluorescent proteins, improving neurosurgical accuracy as a guiding tool. Deleting pathogenic genes, e.g., BACE-1, can facilitate microglial polarisation towards neuropathology-ameliorating states (see Fig. 1).

### Re-engineered microglia in glioblastoma

#### Engineered rAAV2-IL-15 microglia modulate the tumour microenvironment

Gliomas are CNS tumours that start in glial cells. Glioma cells closely interact with their surrounding vasculature and immune counterparts, creating an immunosuppressive microenvironment advantageous for tumour growth [30]. However, in an enriched glioma microenvironment, anti-tumour microglia have a pro-inflammatory phenotype producing increased levels of IL-15 and enhancing the cytotoxicity of proximal natural killer (NK) cells [30]. IL-15 is a cytokine that signals through CD122/CD132 and induces NK cell proliferation.

To simulate the effects of enriched environmental cues, Mormino et al. engineered microglia to produce more IL-15 to inhibit glioma development [31]. Microglia were infected by recombinant adeno-associated virus serotype 2 (rAAV2) carrying IL-15 genes to express and secret IL-15 molecules. rAAV2, non-enveloped and single-stranded DNA vectors, are advantageous in their efficient transduction and minimal cytotoxicity in microglia, thus yielding optimal microglial expression of IL-15 [32]. Cultured microglia are resistant to non-viral conventional transfection methods (e.g. chemical or electrical transfection) so rAAV2 overcome this barrier [32].

In vitro co-culturing of microglia and NK cells demonstrated that microglia-derived IL-15 mainly increased NK cell viability but had little effect on NK cell activation, as indicated by the increased number of NK cells but the unchanged proportion of CD69 + and NKG2D + NK cells [31].

In vivo studies in glioma-bearing mice showed that intranasally delivered rAAV2-IL-15 microglia could infiltrate and accumulate in the glioma mass [31]. NK cells were recruited, as a result, and the glioma volume shrank [31]. Moreover, pro-tumour Arg1^+^ microglia reduced, and rAAV2-IL-15 microglia became more branchy and far-reaching [31]. This indicated the positive modulatory effects of engineered microglia on the tumour microenvironment, making it more favourable for the anti-tumour phenotypes of immune cells. Overall, these preclinical results demonstrated rAAV2-IL-15 microglia’s potential as a treatment for glioma patients. However, this study can be extended and improved.

As rAAV2 cannot penetrate the BBB to reach gliomas, microglia cannot be transduced in vivo [33]. Although culturing and re-engineering microglia in vitro are demanding and laborious, the quality and safety are easier to manipulate and control. Other rAAV serotypes (e.g. rAAV 6, 8, 9) can also efficiently transduce cultured microglia without eliciting aberrant immune response [32]. They, except for rAAV2 and rAAV5, can cross the BBB efficiently to deliver therapeutic genes in the neonatal mice CNS; yet, their targets are limited to neurons and astrocytes as microglia are refractory to rAAVs in vivo [33]. Nonetheless, Rosario et al. overcame this by constructing a modified rAAV6 capsid (TM6) that can transduce microglia efficiently and selectively by (1) a triply mutated Y731F/Y705F/T492V form; (2) a self-complementary genome; and (3) microglia-specific promoters (F4/80 or CD68) [34]. All these modifications improved rAAV6’s specific tropism for microglia and their microglia-selective expression. When intracerebroventricularly injected, this modified rAAV6 enabled significant CD68 promoter-driven expression of the pro-inflammatory IL-6, altering the immune activation state in mice brains in vivo [34]. TM6 might be capable of in vivo transduction of microglia with IL-15 to treat gliomas, but more experimental evidence is required.

Besides IL-15, a more upstream target can be nuclear receptor subfamily 4 group A member 2, which is upregulated in microglia contributing to a pro-tumorigenic tumour microenvironment in glioblastoma [35]. Microglia-specific knockdown of this gene rewired microglia, making it more anti-tumorigenic [35]. More promising targets are warranted to improve glioblastoma by genetically engineered microglia.

#### BV2 microglia, stimulated by nanoparticles and loaded with fluorescent dyes, guide brain tumour surgery

Engineered microglia, besides altering the microglial genetic expression to secret therapeutic molecules at targeted sites, could be utilised to improve the precision for surgical resection of brain tumours to minimise neurological deficits. Guo et al. engineered murine BV2 microglial cells, DiDBV2-Fe, by stimulating them with citric-acid coated iron oxide nanoparticles (CIONPs) and loading them with near-infrared fluorescent dye DiD [36].

CIONPs stimulated the BV2 microglial cells to adopt a pro-inflammatory phenotype, as indicated by the upregulated TNF-$\alpha$ (anti-tumour marker) and downregulated pro-tumour markers (Arginase-1 and CD206) revealed by real-time PCR [36]. Furthermore, multiple genes involved in increasing the blood–brain barrier (BBB)’s permeability (e.g., apoE and transferrin) were upregulated, indicating their enhanced transmigration efficiency [36]. Little cytotoxic effects were shown, as the cell morphology and viability remained unaffected by the CIONP concentrations [36]. CIONPs-activated BV2 microglial cells demonstrated a better uptake of DiD. Overall, these engineered BV2 microglial cells were satisfactory in vitro.

In vivo imaging of mice bearing human glioblastoma cells demonstrated that (1) intracarotid artery injection of activated DiDBV2-Fe better accumulated in the brain than when intravenously injected; (2) DiDBV2-Fe had a higher biodistribution and accumulation in the brain than the commercial intraoperative optical imaging agent 5-ALA; (3) confocal microscopy and immunofluorescent imaging showed a better co-localisation of fluorescent signals and tumour markers, indicating the high tumour-targeting specificity of DiDBV2-Fe; (4) DiDBV2-Fe could be attracted towards gliomas, further increasing the specificity, due to tumour’s higher expression of chemotactic factors (CCL2, CCL3, CCL4), compared with normal brain tissues, and upregulated microglial chemotactic receptors (CCR2, CCR4), as revealed by real-time qPCR analysis; (5) preliminary safety assessment of DiDBV2-Fe showed no acute liver injury, phototoxicity, and hypersensitivity reactions, suggesting them as safe intraoperative optical imaging agent vehicles [36]. Engineered microglia can help fluorescence-guided resections of brain tumours achieve more accurate tumour cell identification and optimal outcomes.

Besides genetic insertion, microglia can be used as a biomimetic vehicle for therapeutic delivery. The drug loading is mainly phagocytosis mediated. Strictly speaking, they do not involve alterations in microglial genetic sequence but involve modulation in genetic expression. The two examples below should belong to the next session about therapeutic modulation, but given their common application in glioblastoma/gliomas, they are grouped under this session for reference convenience.

#### Drug-loaded microglia target the tumour through vesicles and nanotubes

Microglia are ideal transport vectors for drug delivery to gliomas, thanks to their capability of phagocytosis-mediated drug loading, and transmigration across BBB to reach the targets within CNS. Du et al. engineered microglia by inducing them to phagocytose liposome-encapsulated paclitaxel (PTX), a chemotherapy drug for glioma treatment, through an “eat-me” signal (dipalmitoyl phosphatidylserine decorating the liposome surface) [37].

In vitro, PTX-loaded microglia tracked and perforated gliomas cells significantly. They demonstrated strong tumour-penetrative ability and inhibitory effects on tumour growth in the gliomas spheroids [37].

In vivo, PTX-loaded microglia transmigrated towards gliomas across the blood–brain barrier and showed positive anti-tumour effects in mice [37]. The tumour growth was inhibited with minimal side effects, as drug-loaded microglia delivered the therapeutic PTX into glioma cells via extracellular vesicles and tunnelling nanotubes, which did not exist between microglia and other brain cells. Moreover, these engineered microglia promoted an anti-tumour microenvironment by increasing CD86 antigen presentation costimulatory molecule and TNF-$\alpha$, a pro-inflammatory cytokine, and decreasing immunosuppressive regulatory T lymphocytes [37]. Overall, these preclinical results demonstrated PTX-loaded microglia’s potential as a treatment for glioma patients.

Besides dipalmitoyl phosphatidylserine, microglial phagocytosis of liposome-encapsulated PTX can be enhanced by glycan ligands of CD33 (CD33L) on the liposome surface [38]. PTX works by disrupting the normal function of microtubules during mitosis to suppress glioma cells’ proliferation. Nonetheless, PTX itself is neuropathogenesis-prone and cytotoxic for microglia: PTX induced peripheral neuropathy, including microglia dysregulation in the spinal cord [39, 40]; induced cognitive impairment by increasing neuronal necroptosis and decreasing synaptic plasticity [41]; and directly activated astrocytes, producing acute pain by the release of TNF-α and SDF-1 (stromal-derived cell factor 1). Although microglia are protected by the liposomal separation from PTX to maintain normal physiological functions, the targeting efficacy of PTX-loaded microglia towards gliomas cells is not secured: how to ensure PTX-loaded microglia only interact with targeted tumour cells? When PTX is delivered in vesicles, not by “safe” nanotubes, how to ensure that they, extracellularly released, are not taken up by other nearby cells (e.g. tumour-associated immune cells)? It is thus suggestible to increase microglia’s gliomas targetability by, for instance, enhanced expression of chimeric antigenic receptors sensitive and specific for tumour-associated antigens. Moreover, a low dosage is a common problem for all biomimetic carriers, including microglia. Henceforth, future work can focus on specificity and dosage improvement.

#### A virus-mimicking nucleic acid nanogel reprograms microglia and macrophages for glioblastoma therapy

Apart from directly phagocytosing anti-tumour therapeutics, microglia taking up reprogramming materials can initiate a wide range of anti-tumour activities. MicroRNAs (miRNAs) are short and non-coding RNA that regulate post-transcriptional expression, mainly involved in RNA silencing-repression of target gene expression. MiR-155 was revealed to downregulate anti-inflammatory cytokines and upregulate pro-inflammatory mediators. Thus, increasing miR-155 levels promotes microglia to adopt an anti-tumour phenotype.

Utilising microglia’s phagocytotic activities, Gao et al. designed Vir-Gel, a virus-mimicking membrane-coated microRNA-155 (miR-155) nanogel, to reprogram microglia from pro-tumour phenotype to anti-tumour phenotype for glioblastoma treatment [42]. To deliver therapeutic miR-155, which is subject to degradation, into the microglia, the delivery vesicles, Vir-Gel, are specially designed. First, to protect miR-155 and extend their circulation time, miR-155 are embedded in the nanogel coated by the erythrocyte membrane. Second, to increase their specificity and targetability, the erythrocyte membranes are modified with two functional peptides: (1) M2pep, which specifically targets microglia; and (2) influenza virus-derived HA2, which promotes the merging of the erythrocyte membrane and microglial endosomal membrane, facilitating the release of miR-155-loaded nanogel into the cytoplasm. Cytoplasmic ribonuclease H can digest the nanogel to release miR-155, re-engineering microglia to adopt the anti-tumour type.

In vitro, flow cytometry and quantitative analysis demonstrated that Vir-Gel enabled the highest uptake of miR-155 by microglia [42]. Furthermore, microglia reprogrammed by Vir-Gel expressed the highest levels of iNOS (anti-tumour phenotype marker) and the lowest levels of CD206 (pro-tumour phenotype marker) [42]. This indicated Vir-Gel’s strong capability in reprogramming pro-tumour microglia into anti-tumour phenotypes.

In vivo pharmacokinetic experiment showed that miR155 delivered by Vir-Gel, intravenously injected, had the longest half-life (~ 10 h) in the blood [42]. In vivo fluorescence imaging of glioma-bearing C57/BL6 mice showed that Vir-Gel enabled the fastest, highest, and most persistent accumulation of miR155 in the brain [42]. Immunofluorescent staining confirmed the co-localisation of miR155 and Iba1 (microglial activation marker) signals, proving miR155 specifically target microglia in the brain. Glioma’s growth was inhibited, and the glioma-bear mice’s survival time was elongated [42]. Flow cytometric analysis of microglia showed a significantly increased proportion of anti-tumour microglia, along with increased pro-inflammatory cytokine IL-12 and decreased anti-inflammatory cytokine IL-10 [42]. This indicated Vir-Gel’s immunomodulatory effects. In summary, all these results indicated miR-155 loaded Vir-Gel could reprogramme microglia into anti-tumour phenotype for glioma treatment.

Gliomas–microglia interaction is bi-directional, and miRNA reprogramming of microglia is increasingly gaining attention. Gliomas cells secret vesicles containing miR-21, which are taken up by microglia to downregulate mRNA targets and increase proliferation after the downregulation of *Btg2 (a tumour suppressor)*, shaping an immune microenvironment favourable for gliomas progression [43]. Mimicking gliomas-derived miR-containing extracellular vesicles, Vir-Gel reprograms microglia into an anti-tumour state by miR-155 delivery. Besides miR-155, miR-124 is the other well-studied miRNAs in microglia that are responsible for the maintenance of the “resting” state [44]. Upregulation of miR-124 decreased the release of pro-inflammatory mediators, but their role in gliomas remained unclear [45]. A large variety of miRNAs have been implicated in the activation and polarisation of microglia and are possible targets for glioma treatment [46]. With further improvement, Vir-Gel might have the capacity to deliver a combination of different miRNAs into microglia that optimise final effects.

In summary, four examples of how re-programmed microglia can be utilised to treat glioma/glioblastoma have been summarised and discussed. The engineered microglia can not only deliver therapeutics and facilitate neurosurgical procedures but also be re-programmed by viral transfection and by phagocytosis of microRNA-loaded nanogel. In vitro results in cell culture and in vivo outcomes in mice have so far been positive. In the future, more effective re-programming methods might accelerate the approval of applying engineered microglia to treat CNS tumours.

### Re-engineered microglia in neurodegenerative diseases

Neurodegenerative diseases (NDs) are featured by neurodegeneration, the progressive loss of neurons structurally and functionally. NDs are affecting millions of people worldwide and encompass a wide range of diseases, two most common of which are Alzheimer's disease (AD) and Parkinson's disease (PD). Microglia plays a significant neuroimmune role in the neurodegeneration [47–49]. Genetically microglia have therapeutic potential for NDs. Plasschaert et al. re-engineered microglia-like cells with lentiviral vectors encoding either codon-optimised human beta-glucocerebrosidase (GBA), or codon-optimised human progranulin (GRN) [50]. These cells expressing GBA and GRN are engrafted in murine models to ameliorate GBA deficiency-associated PD and GRN deficiency-associated frontotemporal dementia (FTD), separately [50]. Compared to enhancing GBA/GRN expression in neurons, re-engineered microglia cause smaller disturbances in neuronal activities. GBA gene therapy improved α-synucleinopathy of midbrain dopamine neurons associated with PD [51] and GRN ameliorated microglial pathology associated with FTD [52]. Moreover, the selective reduction of GRN expressed by microglia in Aβ-mice worsened AD symptoms [53]. Therefore, increasing GRN expression in microglia by genetic engineering might also be a potential therapy for AD. Apart from genetic insertion, genetic deletion in microglia can also treat AD, as elaborated below.

#### Alzheimer’s disease: BACE-1 inhibition facilitates the transition from homeostatic microglia to DAM-1 and decreases Aβ load

Disease-associated microglia (DAM) is a subtype of microglia responsible for maintaining the homeostasis of the brain microenvironment. Stage-1 DAM (DAM-1) is more functional in phagocytosis than stage-2 DAM (DAM-2), which is associated with AD pathology. BACE-1, β-site APP cleaving enzyme–1, is an enzyme heavily involved in Aβ generation. Suppression on BACE-1 enzymatic activities has decreased Aβ load and improved cognitive functions in AD adult mice (5xFAD) [54]. Clinical phase II/III trials of BACE inhibitors, targeting selective active sites, are also ongoing [55].

Plasschaert et al. discovered that BACE-1 deletion in microglia induced a transition to DAM-1 phenotype and elevated multiple signalling pathways (e.g. PI3K/AKT, IL-6, and Rho), promoting more efficient microglial phagocytosis of Aβ. The tamoxifen-inducible microglia-specific BACE-1 depletion was accomplished by breeding microglia-specific Cre-carrying mice with BACE-1 conditional mice carrying loxP-flanked genes. Furthermore, BACE-1 influenced TLR/IL-1 signalling to manipulate PI3K–AKT–Rac1 activity in microglia, thus regulating the uptake of amyloid plaques. BACE-1 reduction thus increased Aβ uptake. As Aβ likely causes AD when accumulated abnormally, engineering microglia by targeted inhibition of BACE-1, which effectively reduces Aβ load, may present a therapeutic strategy for AD treatment.

However, BACE-1 inhibition might lead to APP (substrates of BACE-1) elevation and impair BACE1-mediated processing of endogenous CHL1 (cell adhesion molecule L1-like protein), exacerbating mechanism-based side effects in, e.g., Down syndrome associated with APP elevation [56]. Moreover, complete knockout of BACE1 resulted in deleterious phenotypes in the mouse models, and even partial deletion could induce long-term potentiation (LTP) deficit in hippocampal CA1, raising concerns about BACE-1 inhibition’s safety and tolerability [57]. It is, however, unclear whether targeted inhibition/deletion of BACE-1 in microglia exerts similar side effects. An alternative to BACE-1 inhibition for AD treatment is considerable. Kim et al. suggested the restoration of GGA3 function [56]. GGA3, Golgi-localized γ-ear-containing ARF binding protein 3, is responsible for trafficking BACE1 towards lysosomes for degradation. Its reduction is related to BACE elevation in the post-mortem brains of AD patients. Therefore, enrichment of GGA3 in microglia can be an alternative to direct BACE-1 depletion, minimising potential side effects of APP accumulation. More studies are required to examine the potential side effects of targeted inhibition/deletion of microglial BACE-1.

### Re-engineered microglia in neuropsychiatric diseases

#### Inflammation-related depression: photoresponsive vaccine-like CAR-M-UZPM system

Depression is an emotional disorder characterised by a persistent feeling of pervasive low mood and low self-esteem, and aversion to normally enjoyable activities. Neuroinflammation, mainly induced by microglia activation, has been indicated in the pathophysiology of depression. Recent studies suggested depression as a microglial disorder, given microglia’s roles in immune regulation, synaptic plasticity, and neural networking [58]. Microglia derived from depressed female patients demonstrated an increased M1-polarisation and decreased M2-polarisation [59]. Reducing pro-inflammatory (M1-type) microglia might alleviate neuroinflammation-related depression [59, 60].

**Fig. 2:**
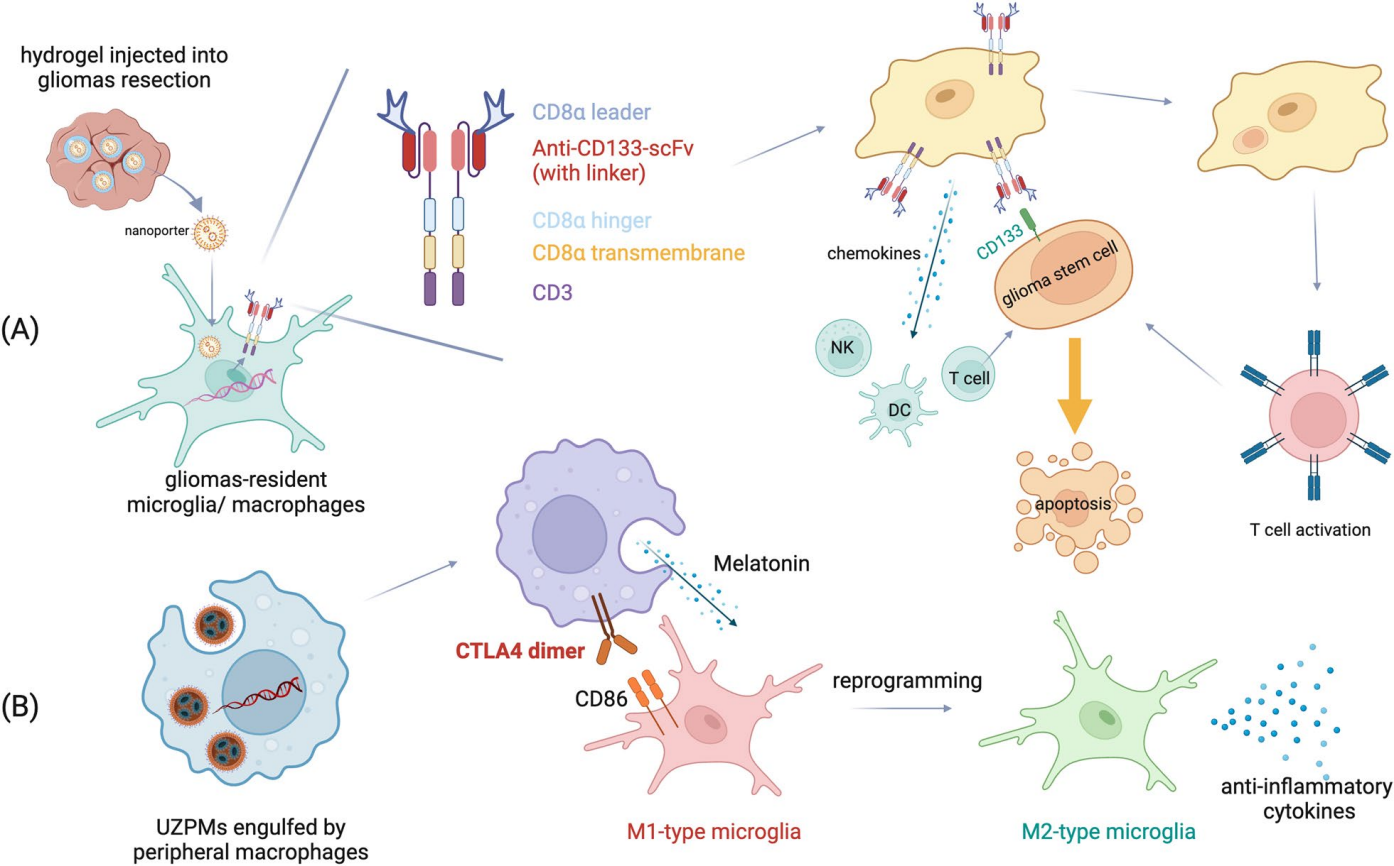
Mechanisms of CAR–microglia/macrophages. **A** Gliomas-resident microglia/macrophages express GSC-specific CARs to eliminate GSC and prevent glioblastoma relapses. **B** UZPMs induce macrophages to express CTLA4 and melatonin to increase M2 anti-inflammatory microglia to alleviate inflammation-related depression. This figure was created with https://www.biorender.com

Liu et al. reengineered macrophages by introducing a light-responsive system, named UZPM, and modifying their cell surface with the chimeric antigen receptors (CAR) to target CNS M1-type microglia, specifically [61]. The UZPM system, NaYF4:Yb, Tm (UCNP)@zeolitic-imidazolate framework (ZIF-8)-photoacid (PA) + melatonin (MT), is responsive to 980 nm near-infrared (NIR) light, which excited PA to disrupt ZIF-8 to release the immunomodulatory MT [61]. Furthermore, the system surface is modified with polyethene glycol (PEG), improving biocompatibility, and encapsulated by hydroxylamine-labelled liposomes to promote the uptake by macrophages. CAR is an aldehyde-modified cytotoxic T-lymphocyte-associated protein-4 (CTLA-4), which recognises the co-stimulatory CD86 on M1-type microglia to inhibit their inflammatory activation pathways [61]. Synergising the anti-inflammatory effects of MT and CTLA-4, the CAR-M-UZPM system can effectively inhibit M1-type microglia, which release pro-inflammatory mediators, and transform them into anti-inflammatory (M2-type) microglia, serving as a therapeutic strategy against inflammation-related depression (see Fig. 2).

In vitro, the light-controlled release of MT inhibited pro-inflammatory responses induced by lipopolysaccharides (LPS), derived from Gram-negative bacterial cell walls and recognised by toll-like receptors 4 to elicit downstream signal cascades, in cultured mice microglia cell lines [61]. MT rescued the cell viability and suppressed the morphological transformation to M1-type, which is short and spindle-like. ELISA results demonstrated no change in pro-inflammatory cytokines (IL-6 and TNF-$\alpha$) and upregulation of anti-inflammatory cytokines (IL-4 and IL-10) [61]. Immunofluorescence analyses confirmed the anti-inflammatory effects of the decreased pro-inflammatory marker (CD86) and increased anti-inflammatory marker (CD206) [61]. RNA-seq analyses revealed a shift in the differential gene expression of microglia, suggesting the polarisation from the M1 to M2 phenotype.

In vivo, these biocompatible CAR-M-UZPMs effectively transmigrated across the BBB to reach the mice brain within 6 h, as indicated by the fluorescent signals detected in the brain and the intact hippocampus. A battery of depressive-like behaviour tests was performed, and LPS-infected mice treated with CAR-M-UZPM showed higher sucrose preference and less immobility in the tail suspension and forced swimming test, suggesting CAR-M-UZPM’s anti-depressive effects [61]. Moreover, they were better at inhibiting depressive-like behaviours than fluoxetine, a traditional anti-depressant drug [61]. Further analyses by ELISA, immunohistochemistry and RT-qPCR revealed that the anti-depression effects were exerted by inhibiting LPS-induced neuroinflammatory response. Finally, whole-cell patch recordings in ex vivo brain slices revealed that intermittent CAR-M-UZPM treatment suppressed LPS-induced overactivation of basolateral amygdala neurons to alleviate depression [61]. Overall, CAR-M-UZPM serves as an ideal drug-delivery platform for depression treatment.

Noteworthy, this study is not expressing CAR on microglia but on infiltrating macrophages to target M1-type microglia to treat depression. The CAR in the UZPM system is CTLA-4 that targets M1-type microglia and polarize them into M2-type microglia, thus alleviating neuroinflammation-related depression. It is novel as CAR is usually expressed on tumour-associated macrophages (CAR-M) for tumour immunotherapy. CAR is specially designed to facilitate macrophages attaching to a specific cancer cell antigen [62]. Compared with other CAR-equipped immune cells, CAR-M is advantageous in penetrating solid tumours and clearing malignant tissues by phagocytosis [63]. CAR-M has been suggested for treating glioblastoma, an aberrant brain tumour, by improving the inflammatory microenvironment for tumour control [64]. The diversity of CAR and the light-responsiveness of UZP make macrophages a versatile and controllable platform for therapeutic delivery to the CNS to treat different neurological diseases, besides depression and glioblastoma.

Microglia and macrophages expressing CAR serve as an effective postoperative glioblastoma therapy (see Fig. 2). Post-surgery residual glioma stem cells (GSCs) have been difficult to track and eradicate, leading to glioblastoma propagation and quick recurrence. Chen et al. generated CAR-microglia/macrophages by injecting a hydrogel, with the CAR gene-laden nanoporter, into the cavity of tumour resection. As CD133 is a marker for GSCs, the CAR is mainly composed of an anti-CD133-scFv, specifically targeting GSCs, and a CD8α, which presents processed GSC antigens to activate T cells, with CD3 being intracellular costimulatory domains [65]. These CAR–microglia/macrophages specifically engulf GSCs and induce T-cell-based adaptive immune response towards GSCs, and they secret chemokines that recruit other immune cells (NK, DC, T cells) to trigger the GSC apoptosis [65]. CAR–microglia/macrophages in preclinical humanised mouse models successfully prevented postoperative relapse, representing a promising glioblastoma treatment, and further clinical trials are warranted. Nonetheless, it remains unclear if CAR–microglia and CAR-macrophages are performing the same, due to the difficult distinguishment between microglia and macrophages, and a lack of experimental evidence for comparison—CAR-M is much less developed than CAR-T therapy in treating glioblastoma, and gliomas-associated macrophages were only used to deliver interferon-alpha to improve the efficacy of CAR-T cells [66].

Although microglia are generally defined as CNS-resident macrophages, macrophages, derived from blood-borne monocytes infiltrating the brain and used to treat glioblastoma (brain tumour), are not the same as CNS-resident microglia in several aspects. First, they have different origins: microglia are derived from the yolk sac and migrate to the brain early in embryonic development, while macrophages are derived from the myeloid lineage of hematopoietic stem cells. Tissue-resident macrophages originate from monocytes in the bloodstream that enter and differentiate in specific tissues. Second, they present different markers: microglial markers include P2Y12, TMEM19, TREM2, low CD45, and microglia are Pu.1 dependent; macrophages’ markers are high CD45, CD44, CD169, and they are Myb dependent [67, 68]. Furthermore, macrophages engrafted in the brain parenchyma acquire microglial characteristics over time but still differ from microglia in transcriptomic profiles, chromatin accessibility landscapes, and the response to challenges, despite prolonged CNS residence [69]. They are thus functionally distinct. It is thus necessary to further Chen et al.’s study in distinguishing CAR-macrophage and CAR–microglia in terms of transformation efficiency (whether one can express CAR more stably and sustainably than the other), functional efficacy (whether one can better eradicate tumour cells than the other), and safety (whether one is more cytotoxic than the other). Overall, infiltrating blood-borne macrophages and CNS-resident microglia are similar but different in several aspects, and more studies are warranted to unravel the differences between CAR-macrophages and CAR–microglia in treating neurological diseases.

### Re-engineered microglia in autoimmune diseases of the central nervous system

Autoimmune diseases arise from the abnormal immune response to functioning normal cells. The immune system mistakenly recognise “self” antigens as “non-self” antigens, initiating destructive attacks that impede the normal functioning of specific body organs. There are more than 80 autoimmune disorders, around 30 of which happen in the nervous system [70]. Multiple sclerosis (MS) is a CNS autoimmune disease characterised by dysfunctional myelination and neuronal damage, in which microglia are involved in both immune response and demyelination [23, 71]. Given their extensive participation in the pathogenesis of MS, microglia have been a therapeutic target for MS treatment.

#### NT-3 expressing microglia

CNS repair remains difficult due to the pro-inflammatory microenvironment and limited accessibility of BBB-impenetrable therapeutics. Neurotrophin-3 (NT-3) is a well-studied trophic factor for neural survival, development, and plasticity [72]. In the adult brain, ramified resting-state microglia rarely express NT-3 [73]. Activated microglia produce NT-3 and its receptor TrkC in the injured MS CNS. NT-3 regulated proliferation and phagocytosis in the microglia [73] and functioned as an anti-inflammatory suppressor of microglial activation, reducing the release of inflammatory iNOS, NO, and TNF-α [72]. Moreover, neuron-derived NT3 inhibit MHCII inducibility, which is highly expressed during neuroinflammation and the neurodegenerative process, of microglia by binding to the p75 neurotrophin receptors [74]. Therefore, endogenous NT-3 plays a neuroprotective role in MS. Yet, it is insufficiently expressed in the MS brain, necessitating the supplement from exogenous sources.

Beutner et al. engineered microglia derived from embryonic stem cells (ESdM) to express therapeutic neurotrophin-3 (NT-3), which transmigrated across the BBB to reach the inflammatory target and promoted the repair of CNS lesions in experimental autoimmune encephalomyelitis (EAE), a mouse model of multiple sclerosis [75]. Before transducing ESdM with the mouse NT-3 gene, a lentiviral vector was used to carry the gene, downstream of the phosphoglycerate kinase (pgk)-promoter. The cytokine profile of EsdM transduced with NT-3 remained unchanged.

Intravenously transplanted EsdM could only migrate into the inflammatory spinal cord of EAE mice but not healthy mice [75]. Afterwards, fluorescence imaging and flow cytometry analysis revealed the presence of CD45 or Iba1 (microglia markers) and Ki67 (proliferative marker) loss proximal to the inflammatory lesions, indicating transplanted ESdM’s adaptation towards a resident microglial phenotype [75]. NT-3 provided by EsdM improved the clinical symptoms significantly [75]. The researchers also observed ameliorated demyelination, reduced axonal injury, and enhanced anti-inflammatory cytokine milieu in EAE mice treated with NT-3-EsdM. In vitro culture further validated NT-3-EsdM’s pro-repair effect by promoting neurite outgrowth. Overall, ESC-derived microglia engineered to express NT-3 seemed promising for repairing the lesioned CNS in neuroinflammation-related diseases, for example, multiple sclerosis and neurodegenerative diseases. In the future, microglia from patient-derived induced pluripotent stem cells, avoiding transplant rejection, might serve as a promising therapeutic vehicle for NT-3 or other drugs to treat human CNS diseases.

However, this study only demonstrated EsdM’s penetrability into the spinal cord but not the brain. Thus, the application of EsdM is currently limited to spinal cord MS, probably due to difficulties in crossing the BBB to reach the brain. Moreover, due to a lack of comparative data, it is not sure if EdsM is more advantageous than other methods available for increasing NT-3 levels, in terms of efficacy and safety. For example, glatiramer acetate (GA), an immunomodulator, could also augment the expression of NT-3 by T cells and resident neurons and astrocytes in the brain [76]. GA injection is much less laborious than EsdM treatment, which requires genetic engineering, quality control, cell culture, and transplantation. Furthermore, for EsdM to enter clinical trials, the problems of immunogenicity and oncogenicity are unignorable concerns.

#### Microglia-derived IL4-containing vesicles

Apart from NT-3, microglia re-engineered to express IL-4 have also demonstrated therapeutic effects in EAE, the mouse model of human multiple sclerosis. It has been reported that microglia increase extracellular vesicles (EVs) and EV-dependent inter-microglia interaction during human multiple sclerosis (MS) and mouse EAE. IL-4, an anti-inflammatory cytokine, showed high efficiency in suppressing neuroinflammation in these models. Casella et al. transfected murine BV-2 microglial cells with lentiviral plasmids carrying the murine IL-4 gene [77]. EVs secreted by these re-engineered microglia contained IL-4 proteins and mRNA, and polarised recipient myeloid to an anti-inflammatory phenotype, in an STAT6-dependent way [77]. To further increase the uptake efficiency of EV, lentiviruses carrying Lactadherin (Mfg-e8) gene, conveying “eat me” signals to phagocytes, were also utilised to infect the microglia [77].

In vivo, IL-4^+^Mfg-e8^+^ EVs injected into the cisterna magna of EAE mice improved clinical symptoms and reduced tissue damage, including demyelination, axonal loss, and inflammatory infiltration [77]. Immunofluorescence analyses revealed a signification reduction of the inflammatory marker, iNOS, and an upregulation of the anti-inflammatory marker, Arg1, in the CNS phagocytes [77]. This suggested the re-engineered microglia-derived EVs were capable of reprogramming in vivo microglia into anti-inflammatory phenotypes to ameliorate pathological neuroinflammation, serving as a therapeutic agent beneficial for MS/EAE treatment.

### Platforms for microglia engineering

Currently, most re-engineered microglia are only tested in cell culture and animal models, which limits their application in treating human neurological diseases. One important step forward is to expand in vitro experiments in human cell lines.

Dolan et al. provided a platform for generating and manipulating human microglial states in vitro, modelling different transcriptional and functional microglial subtypes in different neuropathological contexts [78]. Microglia differentiated from human stem cells (iMGLs) were exposed to neurological challenges (CNS substrates, e.g. synaptosomes, cellular debris, or synthetic Aβ fibrils) to generate extensively diverse transcriptional profiles, analysed by single-cell RNA sequencing (scRNAseq). Most of these iMGLs clusters exhibited transcriptional signatures similar to in vivo human brain microglia [78]. A robust lentivirus-transduction approach was established to enable scalable analyses of microglial functional states. The lentiviral transduction efficiency was improved by co-delivering Vpx, a protein derived from the simian immunodeficiency virus. Vpx, packaged in virus-like particles, can degrade barriers in human microglia that impede lentiviral transduction. To exemplify, the authors utilised this platform to examine the role of enriched MITF (Melanocyte Inducing Transcription Factor), a transcription factor upregulated in the microglia, in the AD context. As a result, they found that MITF led to a neurodegenerative disease-associated transcriptional signature contributory to a highly phagocytic state, revealed by scRNAseq and functional analyses [78]. This demonstrated the platform’s potential in unravelling the function of different microglial states and their roles in homeostasis maintenance and different neurological diseases. More recently, another platform has been created to uncover regulators of these states.

M. Dräger et al. presented a CRISPRi/a platform to screen regulators of microglial states in vitro, possible therapeutic targets, and systemically characterise their functional states [79]. Extensive genetic perturbations, by inducible CRISPR interference (CRISPRi) and activation (CRISPRa), were conducted in microglia derived from human induced pluripotent stem cells. Single-cell RNA sequencing then systemically elucidated the functional consequences of these genetic perturbations to uncover state-associated genes and regulators of these states [79]. This platform could screen modifiers of (1) microglial survival and proliferation; (2) microglial activation; and (3) synaptosome phagocytosis. More importantly, it could uncover regulators of microglial states, for example, colony-stimulating factor-1 (CSF-1R) inhibition is correlated with selective depletion of the osteopontin^+^ (SPP1^+^) state disease-associated state [79]. Another recently available CRISPR-based platform deploys the electroporation of Cas9 ribonucleoproteins and synthetic DNA repair templates to genetically modify microglia [80]. This platform is advantageous in being rapid and reduced in off-target effects given the absence of viral or plasmid vectors and short half-life of electroporated proteins [80].

In comparison, the former platform more fully recapitulates different neuropathological contexts, while the other is a more suitable method to screen microglial regulator genes. However, the latter platform enables more large-scale screens and avoids many variables introduced when creating the neurological challenges to model different neuropathological contexts. Moreover, CRISPR is a more efficient and less laborious genetic editing tool than lentivirus transduction, which further requires the facilitation of Vpx to overcome microglial barriers. In general, both platforms provide robust protocols for the derivation of microglia from stem cells, enabling a wide variety of future applications: (1) using patient-derived iPSCs can help identify potential disease-specific therapeutic targets of microglia; (2) introducing these microglia into 2D cocultures or 3D brain organoids enables the investigation of inter-cellular interactions (e.g. neuron- and astrocyte- microglia interrelationship); and (3) transplanting microglia into humanised animal models enables the study of in vivo human microglial signature.

In the future, genes pivotal for the switching of microglial polarisation, e.g., MSX3 [81], KCa3.1 [82], and CSF-1R [3] will be the important targets of genetic editing by efficient tools, such as CRISPR–Cas9. Genetic targeting of these key genes might serve as an efficient strategy for microglial manipulation in different neuropathological contexts.

Although genetic reengineering is a potent way to switch microglial polarisation, it can be laborious, irreversible, and might bring unexpected side effects. Therapeutic modulation is another strategy to reversibly change microglia’s functional states, by modifying genetic expression without altering the genetic composition of microglia.

## Modulating microglia in the central nervous system

Most of the therapeutic modulation is achieved by binding to different receptors on the surface of microglia, which triggers intracellular signalling cascades leading to microglial polarisation. However, some therapeutics work by regulating the levels of RNA (e.g. miRNA and lncRNA), and some work in a less direct way, for example, by intercellular interactions (see 3.4.). This session introduces the major categories of the microglial receptor, summarises different kinds of therapeutic modulators (see 3.1.), and discusses how nanoparticles and extracellular vesicles can be excellent vehicles to improve the delivery efficiency of these therapeutic molecules (see 3.2. and 3.3.) (see Fig. 1).

### Receptors on microglia: modifiable targets of neurological diseases

Microglia express a myriad of receptors on the surface. Different ligand–receptor interactions can activate or inhibit microglia in different actions, including microglial phagocytosis or inflammatory responses or both, depending on different neuropathological contexts [21].

*Some are only involved in the release of proinflammatory mediators* Take excitatory RAGE and inhibitory CD200R1 for illustration. RAGE are receptors of advanced glycosylation end products, which interact with neurotoxic proteins, e.g. A $\beta$, to activate NF-kB signalling pathways in microglia, leading to transcription of pro-inflammatory cytokines [83]. Blocking the A $\beta$-RAGE interaction could reduce oxidative stress in vitro, but in vivo effects in AD were not satisfactory [84]. RAGE level is higher in PD, whose activation leads to MPTP/MPP + -induced NFkB-mediated dopaminergic neuronal death [85, 86]. The S100B (a calcium-binding protein)–RAGE interaction exacerbates demyelination by impairing myelin repair and oligodendrogenesis in MS and has been implicated in AD [87, 88]. Some inhibitory receptors, such as CD200R1, recognise CD200 secreted by neurons and other glia, leading to a reduction in neurotoxic pro-inflammatory response and attenuation in microglial activation [89]. Reduced expression of CD200R1 elevated risks in PD [90, 91]. Its reduction has been also indicated in AD and MS [91, 92]. Thus, RAGE and CD200R1 might be promising targets for neurodegenerative diseases.

*Some are only involved in the phagocytosis of misfolded proteins* SR-A1, TREM2, and TAM receptors are implicated in the pathogenesis of AD. SR-A1, type-1 scavenger receptor A, is a trimeric receptor that recognises various ligands and is expressed by activated microglia. It recognises A-beta and promotes microglia to Internalise and reduce A-beta, making it a possible therapeutic target for AD [93]. TREM2 is a single-pass transmembrane receptor for anionic ligands, involved in the TREM2–DAP12–DAP10 signalling cascades, which enable microglia to fully become disease-associated microglia (DAM) to engulf amyloid plaques and provide neuroprotection [94, 95]. Loss-of-function variants (R47H) in the TREM2 gene increase the risk of developing AD in humans [96]. Finally, microglia express TAM receptor tyrosine kinases Axl and Mer, which make microglia mediators of recognition and endocytosis of amyloid plaques [97]. TAM-driven microglial engulfment promotes dense-core plaque development in the AD pathogenesis [97].

Furthermore, microglial phagocytosis of neurons involves three sets of signalling systems—“find-me”, “eat-me”, and “don’t-eat-me” signalling, as summarised in Butler et al.’s review [98]. Neurons releasing ATP/ADP and CX3CL1 will attract microglia to come to proximity by the chemotaxis-triggering ADP–P2Y12 and CX3CL1–CX3CR1 interactions [99, 100]. The activation of microglial phagocytosis requires another set of ligand–receptor interactions. UTP/UDP released by neurons will be detected by P2Y6 receptors on microglia, and neuronal membrane-bound phosphatidylserine can activate microglia by binding to TREM2/GPR56 and MERTK/TREM2/VNR/MEGF10 [98]. Desialylated proteins/lipids on the neuronal surface bind to MERTK/LRP1/CR1/3/4 to trigger microglial phagocytosis of neurons; however, after sialylation, these ligands, in turn, bind to SIGLEC to inhibit the phagocytosis [101]. The other known “don’t-eat-me” signal has been the neuronal CD47–microglial SIRP $a$ interaction [102]. The outcome of microglial phagocytosis of neurons is an orchestration of these multiplexed signalling.

*Finally, many more microglial receptors are involved in the triggering of both* They include complement receptors, Fc receptors, toll-like receptors (TLR), and so on. Complement receptors are soluble and membrane-associated proteins promotive for opsonisation and inflammation. Fc receptors recognise the constant domains (Fc) of immunoglobulins (Ig) secreted by B lymphocytes. For example, they mediate microglial phagocytosis of A-beta by recognizing the Fc domains of anti-A-beta antibodies and activate microglia by infiltrating IgG due to BBB with increased permeability [103]. Thus, they are implicated in the progression of AD. Finally, toll-like receptors on microglia are involved in a wide range of neurodegenerative diseases. For instance, TLR2 recognises exosomal a-synuclein to activate the microglia [104]. Yet, over-activation due to excessive a-synuclein phagocytosis might result in the microglia–neuron transmission of a-synuclein; in contrast, TLR4 enables the clearance of a-synuclein via TLR4-NF-κB-p62-mediated synucleinphagy [105]. Modulating TLR2 or maintaining the TLR4-mediated synucleinphagy might confer neuroprotection for PD.

Microglial response, following different ligand–receptor binding, is much more multiplexed than the abovementioned. Microglia not only secrete pro-inflammatory mediators but also destructive enzymes, such as matrix metalloprotease 3 (MMP3), when they become hypofunctional after phagocytosing live neurons with P301S mutational tau aggregates [106]. Depending on the different states of microglia and their microenvironment, they also release anti-inflammatory mediators. Having a more comprehensive understanding of these microglial receptors and the corresponding response in different neuropathological contexts will greatly facilitate the development of engineering microglia for the treatment of various neurological diseases.

### An overview of therapeutic modulators of microglia

Most of the therapeutic modulators for microglial modulation are proteins, while some are acidic molecules. Some, e.g., cytokines, work as ligands and bind to the receptors on the surface of microglia to activate certain downstream signalling cascades. Some work as antibodies or inhibitors to block some of the receptors involved in microglial phagocytosis or release of inflammatory mediators, inactivating related signalling pathways. Other therapeutic modulators work by inactivating some RNA molecules associated with aberrant microglia activation. Given the large variety of therapeutic modulators and neurological diseases involved, they are summarised in Table 1, in an organised way, for easy reference.

**Table 1:** Therapeutic molecules for microglia modulation in different neurological disease models

	Microglial targets	Disease models	Animals	Working mechanisms and effects on microglia	Refs.
IL-12	IL-12R	Glioblastoma multiforme	Rat	Long-lasting expression of IL-12 increased infiltration of activated microglia to the tumour, inhibiting the tumour growth and increasing the survival of tumour-bearing rats	[107]
(Astrocytic) IL-3	IL-3R	Alzheimer’s disease	5xFAD mice and human samples	Aβ deposits induced microglia to upregulate IL-3Rα, the receptor for IL-3, to become more responsive to IL-3, constitutively produced by astrocytes. The IL-3–IL-3Rα interaction induced reprogramming in microglia, enhancing their ability to cluster and clear misfolded protein aggregates (e.g. Aβ and tau). In vitro, the pathology and cognitive functions in AD mice were improved. Increasing IL-3 or upregulating IL-3Rα expression might be promising therapeutic targets for AD treatment	[108]
IFN-γ	IFN-γR	Alzheimer’s disease	AD transgenic mice	Chronic exposure to Aβ induced metabolic defects in microglia, making them tolerant to Aβ. Chronic IFN-γ treatment restored the immunological functions of microglia, and mitigated AD-like pathology and improved cognitive functions in mice	[109]
Anti-pyroglutamate-3 Aβ antibodies	pyroglutamate-3 Aβ	Alzheimer’s disease	Mice	Pyroglutamate-3 Aβ (pGlu-3 Aβ) is a post-translationally modified Aβ species associated with Alzheimer’s disease (AD). Anti-pGlu-3 Aβ antibodies resulted in more plaque-associated microglia for Aβ clearance	[110]
TREM 2 antibodies	TREM2	Frontotemporal lobe degeneration	Grn KO mice (progranulin deficiency model)	Antagonistic TREM2 antibodies enhanced TREM2 shedding, reducing its signalling in microglia. As a result, microglial hyperactivation and phagocytic activities were ameliorated. However, this did not attenuate neurotoxicity. It turned out that TREM2-dependant microglia hyperactivation was neuroprotective. TREM2 agonists might be a therapeutic strategy in this pathological context	[111]
Anti-miR-155 inhibitor	miR-155	Amyotrophic lateral sclerosis (ALS)	SOD1 mice	Targeting miR-155 in SOD1 mice restores dysfunctional microglia and ameliorates ALS symptoms	[112]
LncRNA GAS5 inhibitor	LncRNA GAS5	Multiple sclerosis	EAE mice	LncRNA GAS5 potently inhibit microglial M2 polarisation, by binding to PRC2 to suppress IRF4 transcription. LncRNA GAS5 inhibitors promote M1 polarisation, attenuating EAE severeness and promoting remyelination	[113]
HDAC inhibitors (Scriptaid)	Histone deacetylase inhibitor (HDAC)	Severe traumatic brain injury (TBI)	Mice with TBI	Scriptaid upregulated microglial glycogen synthase kinase 3 beta (GSK3β), which phosphorylated and inactivated phosphatase and tensin homologue (PTEN), thereby enhancing phosphatidylinositide 3-kinases (PI3K)/Akt signalling, an intracellular signal pathway that promotes cell survival and proliferation, and polarizing microglia towards M2. Thus, inhibition of HDACs in microglia is a potential future therapy in TBI and other neurological conditions with white matter destruction	[114]
Chlorogenic acid (CGA)	MIR497HG/miR-29b-3p/SIRT1 Axis	Oxygen and glucose deprivation (OGD)-elicited neuroinflammation	Cell culture	CGA attenuated OGD-mediated neuroinflammation and oxidative stress in microglia and inhibited microglia-mediated neuronal apoptosis. CGA increased the levels of MIR497HG and SIRT1 and suppressed the levels of miR-29b-3p in BV2 and HT-22 cells	[115]
Sialic acid mimetic	CD33	Alzheimer’s disease	Cell culture	The sialic acid mimetic bound to CD33, a cell surface receptor, and increased the uptake of Alzheimer peptide into microglia	[116]

They mainly include four categories: cytokines, antibodies, inhibitors, and acidic molecules

Cytokines: IL-12 is a pro-inflammatory cytokine enhancing both innate and adaptive immune responses towards tumour cells; IL-3 is a pro-inflammatory cytokine that enhances the proliferation and differentiation of monocytes; IFN-γ is a pro-inflammatory cytokine that promotes macrophage activation

Antibodies: anti-pyroglutamate-3 Aβ antibodies recognise and facilitate the removal of pyroglutamate-3 Aβ, an N-terminally truncated and post-translationally modified Aβ isoforms associated with the neurodegeneration in Alzheimer’s disease; TREM2 antibodies recognise and block TREM2, receptors on the microglial surface

Inhibitors: Scriptaid is an enzyme inhibitor (histone deacetylase) that enhances the global acetylation of histones to increase the expression of certain microglial genes

Acids: chlorogenic acid is a polyphenol with antioxidant effects, reducing oxidative stress by scavenging free radicals; Sialic acid are present at the terminal of secreted or membrane-bound glycoproteins and glycolipids

### Delivery of therapeutics-loaded nanoparticles

Nanoparticles are small particles ranging between 1 to 100 nm in size. They can be organic (polymersomes and liposomes) or inorganic (metal or carbon-built nanoparticles), depending on the construction materials. As a drug delivery vehicle, nanoparticles are advantageous in (1) their high surface area to volume ratio enables more effective interaction with target cells; (2) extending the half-life of the loaded drug in the physiological environment, maintaining their activity until reaching the targeted sites; (3) flexible BBB penetrability enabled by different surface modification on nanoparticles, for example, encapsulation by biocompatible exosomes, while some nanoparticles, such as gold nanoparticles, can directly cross the BBB via passive diffusion via ion channels; and (4) controllable release of drugs, minimising possible side effects [117]. Given all these advantages, nanoparticles have been regarded as a promising drug delivery platform to therapeutically target microglia-mediated neuroinflammation, in different pathophysiological contexts.

#### AZ-loaded PS-PEG nanoparticles for ischemic stroke

Polymeric nanoparticles are ideal drug delivery vehicles thanks to their diffusibility and biocompatibility. Azithromycin (AZ) was originally an antibiotic to treat bacterial infections, but AZ is found to provide neuroprotective effects in cerebral ischemia [118], by promoting arginase-mediated anti-inflammatory activity and microglial polarisation towards the M2 phenotype [119]. In an oxygen–glucose deprivation (OGD) slice model mimicking the stroke-related cerebral ischemia, polystyrene‐poly(ethylene glycol) (PS‐PEG) nanoparticles, loaded with AZ, better inhibited the pathologic microglial response to OGD than AZ alone [120]. Activated microglia usually respond to OGD by the polarisation towards the M1 proinflammatory state, which might induce neuroinflammation inhibitory for neuronal recovery [121]. This can be indicated by decreased microglial area, because reactive amoeboid microglia are smaller than their inactivated ramified states. AZ’s effect was exhibited by inhibiting the increase of heterogeneity and circularity in microglial morphology in the cultured cortex and thalamus slices [120]. Thus, PS-PEG nanoparticles carrying AZ endocytosed by microglia can inhibit microglial M1 polarisation, providing neuroprotection in cerebral ischemia.

The microglial uptake and accumulation of AZ are dependent on the disease state and nanoparticles’ physicochemical properties. In this case, the uptake of drug-loaded PS-PEG increased with OGD, and PS-PEG exhibited strong diffusive ability thanks to their small size (< 200 nm), overcoming the limitation of the small pores in the brain extracellular space, enabling them to reach the target microglia to deliver the drug effectively [120]. Furthermore, they improved intracellular trafficking of the therapeutics, utilising microglial phagocytosis for nanoparticle internalisation. Therefore, polymeric nanoparticles are an ideal drug delivery tool for microglia manipulation, given their transportability and affinity for microglia in cerebral ischemia.

Apart from AZ, PS-PEG nanoparticles might be utilised to carry other therapeutic molecules with neuroprotective effects for ischemic stroke. Analgecine (AGC), an analgesic extract from the Vaccinia-inoculate rabbit skin, can promote microglial M2 polarisation by inhibiting NF-κB through the TLR4/MyD88 pathway activated by ischemia [121]. Ginkgolide and bilobalide, major trilactone from the leaves of *Ginkgo biloba*, can inhibit TLR2/4 signalling pathways, polarising microglia towards an anti-inflammatory phenotype [122]. Using PS-PEG nanoparticles as a vehicle, these therapeutical molecules might exert better neuroprotective function against ischemia injury, thanks to enhanced uptake by microglia and increased targeting efficacy that might reduce side effects. However, one major concern is the size limitation. The size of the therapeutic molecule should not exceed the maximum capacity of the PS-PEG nanoparticle, otherwise, it cannot be transported towards microglia. This might be a disadvantage for PS-PEG nanoparticles as some protein-based anti-inflammatory drugs are way too large for them to transport. Overall, PS-PEG nanoparticles are a promising vehicle for small molecules to modulate microglial states, providing neuroprotective effects for ischemic stroke.

#### Sim-loaded PEG–PdLLA nanoparticles for neurodegenerative diseases

Some polymeric nanoparticles are more advantageous in overcoming the poor BBB penetrability and off-target effects of therapeutics. Take PEG–PdLLA (methoxy polyethylene glycol-poly(D,L) lactic acid) diblock co-polymers as an illustration [123]. Loaded with Simvastatin (Sim), an anti-inflammatory drug for neuroinflammation-related neurodegenerative diseases, PEG–PdLLA exerted better anti-inflammatory effects on microglia than Sim alone in suppressing pro-inflammatory factors (nitric oxide) and cytokines (IL-6, and TNF- α) in a neuroinflammation model induced by LPS, a potent activator, derived from Gram-negative bacterial walls, of microglial response [123]. PEG–PdLLA achieved so by several attributes: (1) their polymeric materials are stable in a physiological environment, elongating carried Sim’s half-life; (2) they are capable of protracting the release of biological active Sim; and (3) they specifically target microglia, whose endocytosis-mediated internalisation enhances Sim distribution and exposure [123]. More recently, Emmerich et al. [124] found that dendrimer particles conjugated with immunosuppressive dexamethasone (D-Dex) specifically inhibit microglia reactivity, enhancing neural regeneration in the retina. PEG–PdLLA and D-Dex promisingly demonstrate the potential of polymeric nanoparticles as microglial modulation tools.

Overall, both polymeric nanoparticles improve the delivery of therapeutic molecules into microglia by increasing the targeting specificity and internalisation levels. However, the vehicle capacity of nanoparticles is currently limited to small molecules. To transport therapeutic molecules of larger molecular size and larger amounts, cell-derived extracellular vesicles are an ideal alternative.

### Delivery of therapeutics-encapsulated extracellular vesicles

Extracellular vesicles (EVs), bi-lipid-layer-bound vesicles cellularly secreted into the extracellular space, are another ideal vehicle for therapeutic delivery. EVs are advantageous in their high biocompatibility, low immunogenicity, good capacity, and most importantly, BBB penetrability. Moreover, they offer a high targeting ability to CNS lesions to minimise side effects or off-target effects. Therefore, EVs have been utilised to specifically deliver therapeutics for microglia manipulation.

#### Extracellular vesicles encapsulating Bryostatin-1 inhibit neuroinflammation

In the experimental autoimmune encephalomyelitis, the animal model of multiple sclerosis, Wu et al. used EVs derived from neural stem cells (NSC) expressing the ligand of platelet-derived growth factor A (PDGF-A), called EVPs, to carry bryostatin-1 (Bryo-1) [125]. Bryo-1 is a naturally occurring macrocyclic lactone shown to own anti-neuroinflammation effects in MS and AD [126]. PDGF-A greatly increased the targeting efficiency as its receptor, PDGFRα, is markedly expressed by oligodendrocyte progenitor cells in the demyelinated lesions. In the proximity of demyelinated sites are microglia removing myelin debris and providing pro-growth factors for remyelination during their M2 anti-inflammatory phenotype. Yet, when microglia are in their M1 pro-inflammatory phenotype, they release reactive oxygen species and cytokines that worsen the CNS lesions. EVPs packed with Bryo-1 altered the phenotype of microglia by increasing the proportion of M2 microglia, which greatly limited neuroinflammation and clinical signs, as demonstrated by the reduction of many pro-inflammatory cytokines (IL-17, IL1-β, IL-6, IFN-γ, GM-CSF, and iNOS) and the upregulation of anti-inflammatory cytokines (IL-11 and IL-5) in the spinal cords. Demyelination was ameliorated, and BBB integrity was protected, attenuating excessive microglial activation [125]. Moreover, it overcame the undesirable off-target activity of systemically administrated Bryo-1 thanks to EV’s high targeting efficiency to CNS lesions in EAE mice. Therefore, EVP-Bryo-1 is a potential strategy for microglia modulation in neurological conditions, especially where white matter is destructed and neuroinflammation is uncontrolled.

#### Extracellular vesicles, derived from oligodendroglia and enriched for HSPB8, protect against oxidative stress

Besides NSC-derived EVs, oligodendroglia-derived EVs enriched with heat shock protein B8 (OL-HSPB8-EVs) could reduce oxidative stress in activated microglia [127]. HSPs are molecular chaperones engaged in the regulation of proteostasis, encompassing protein reconstruction and degradation. HSPB8, together with co-chaperone BCL-2-associated athanogene 3 (BAG3), assists in selective autophagy of some proteins to attenuate oxidative stress and thus cell death. As expected, OL-HSPB8-EVs, internalised by microglia cell lines and primary mixed neural culture cells, preserved chaperone activity, and thus selective autophagy, in stressed conditions, alleviating proteotoxic insults from the accumulation of cytotoxic protein aggregates [127]. This further prevents the over-reaction of microglia by misfolded proteins in the pathological contexts of neurodegenerative diseases. Thus, OL-HSPB8-EVs might be applied in various proteinopathies, where misfolded protein aggregates are associated with microglia-mediated neuroinflammation, including, for example, Alzheimer’s disease featured by extracellular A $\beta$ plaques and intracellular fibrillary tau tangles, and Parkinson’s disease with Lewy bodies. However, the above results are only generated from in vitro experiments, the authors did not provide in vivo results so far. It is thus expected to test the ability of OL-HSPB8-EVs to penetrate BBB to reach the brain in vivo, and whether they can exert therapeutic effects in animal disease models as ideal as in vitro experiments. Overall, OL-HSPB8-EVs are a possible tool for microglia modulation in neuropathological contexts, where proteostasis is imbalanced, with more examination warranted.

### Delivery of engineered cells/microbes

#### IL-13-producing mesenchymal stem cells transplanted intracerebrally limit microgliosis

Intercellular interaction between microglia and other engineered cells also has the potential to modulate microglial activities. Le Blon et al. engineered mesenchymal stem cells to express and secret IL-13 and intracerebrally transplanted them in the cuprizone (CPZ) mouse model, an established model for MS [128]. IL-13 is a cytokine primarily secreted by T helper type 2 cells and exerts anti-inflammatory effects. In this study, CX3CR1^+^ microglia will give eGFP fluorescence, discriminated from CCR2^+^ graft-infiltrating peripheral macrophages that give RFP fluorescence. Following CPZ treatment and IL-13-MSC transplantation, all microglia/macrophages were activated, indicated by the activation marker, F4/80, which is not expressed by resting-state microglia in healthy brain tissue. The grafting of IL-13-MSC in the splenium, the thick posterior of the corpus callosum, limited cuprizone-induced microgliosis, indicated by a significant decrease in CX3CR1^eGFP/+^ microglia density in the whole splenium [128]. Furthermore, microglia and infiltrating macrophages around the graft all expressed Arg1, an M2 immunosuppressive marker [128]. Therefore, IL-13 produced by MSC not only recruited anti-inflammatory M2 macrophages, but also limited cuprizone-induced microgliosis, neuroinflammation, and demyelination, improving MS symptoms in the mice.

#### Engineered *Escherichia coli* in the gut provide neuroprotective effects

Long-distance modulation of microglia via the gut–brain axis, microbiome–microglia crosstalk specifically, is also possible [129, 130]. Peripheral inflammation has been a potential risk factor for neurodegenerative diseases, such as PD. Intestinal inflammation was implicated as a driver of the PD pathogenesis [131]. Intraperitoneally injected LPS-induced peripheral inflammation enhanced CNS microglia pro-inflammatory response and nigral dopaminergic neuronal loss in a PD mice model [132]. Probiotics that improve microbiota and therapeutics that suppress intestinal or peripheral inflammation (e.g., curcumin) often provide neuroprotective effects [133, 134].

Wu et al. engineered *E. coli* Nissle 1917 (EcN), a Gram-negative probiotic bacterial strain, to deliver glucagon-like peptide-1 (GLP-1), modulating the gut microbiota to treat PD [135]. GLP-1 is a peptide hormone produced and secreted by intestinal enteroendocrine L cells, functioning as an incretin, which enhances insulin secretion to decrease blood sugar levels. It has thus been regarded as a therapeutic target for diabetes treatment by alleviating insulin resistance. With the occurrence of evidence suggesting a diabetes–PD relationship, GLP-1 has been regarded as a new neuroprotective strategy. In a PD mice model, orally administered engineered EcN delivered the heterologous GLP-1 to restore the disturbed microbiota by increasing the levels of beneficial *Prevotella* while suppressing the relative abundances of *Akkermansia and Oscillospira,* increasing intestinal vulnerability to oxidative stress [135]. The mice demonstrated (1) improved PD symptoms: the motor deficits were ameliorated, indicated by the increased total moving distance in the open-field test and decreased latency time in the pole test; (2) improved neuropathologic damages in the substantia nigra: decreased Iba1-positive microglia and GFAP-positive astrocyte, indicating suppressed microglia/astrocyte activation, reduced synapse-damaging α-Syn density, and rescued tyrosine hydroxylase (TH)-positive dopaminergic neuronal loss; and (3) suppressed neuroinflammation: pro-inflammatory cytokines (IL-1β, TNF-α, and IL-6) were significantly downregulated, achieved by enhancing p-AKT/AKT (protein kinase stimulating cell growth and proliferation) while inhibiting p-IκB-α (inhibitor of NF-κB, transcription factor that increases production of inflammatory mediators), TLR4 (a transmembrane receptor that recognises Gram-negative LPS and triggers a pro-inflammatory response), and p–p65/p56 (interferon-inducible kinase) [135]. Furthermore, EcN-GLP-1 ameliorated permeability and inflammation in the colon of PD mice by elevating the levels of the tight junction proteins and decreasing pro-inflammatory markers, further providing neuroprotection from peripheral inflammation associated with the pathogenesis in PD [135].

In summary, gut EcN-GLP-1 modulated brain-resident microglia in these three aspects:

1. Decreased Iba1^+^ microglia, implicating less microglia activation;
2. Reduced α-Syn, activators of microglia; and
3. Down-regulated pro-inflammatory cytokines and signalling molecules, markers of M1 microglia.

Overall, EcN-GLP-1 demonstrated significant anti-inflammatory effects in both the colon and the substantia nigra, and created a CNS immune microenvironment unfavourable to microglial M1 phenotype, improving PD neuropathology and outcome. However, the cause-and-effect relationship among these three results should be delineated:

1. The α-Syn reduction is a cause of the decreased microglia activation and downregulated pro-inflammatory mediators. Neuron-released α-Syn can activate microglia via the binding to TLR4, inducing IL-1β and TNF-α upregulation [136] and triggering NF-κB-p62-mediated synucleinphagy that clears α-Syn. As a result, with α-Syn reduced, microglia activation is down-regulated, preventing further neuroinflammation and neurodegeneration [105].
2. The exact function of GLP-1 is, therefore, undetermined. If GLP-1 is to facilitate α-Syn clearance, more microglia should be activated, and thus higher levels of pro-inflammatory mediators. Yet, this was contrary to the observed results. It is, thus, likely that GLP-1 provides neuroprotection by suppressing excessive microglia activation, which leads to neuroinflammation damaging to neurons as well, once α-Syn clearance is complete.
3. However, the observed effects are affected by the timepoints when tissue samples are collected and analysed. The closer to the time when mice received EcN-GLP-1 treatment, the more inflammatory the substantia nigra might be. Therefore, increasing sample collection timepoints might help delineate if GLP-1 is pro-inflammatory or anti-inflammatory for the brain. If it is pro-inflammatory, more microglia activation should be observed at early post-treatment timepoints, compared to those PD mice without EcN-GLP-1 treatment.

Despite the inexact working mechanism of GLP-1, the overall treatment outcome in PD mice is positive. Clinical trials in human are expected to examine the therapeutic effects of EcN-GLP-1 in PD patients. In the following years, EcN-GLP-1 might inspire more engineered probiotic platforms for the treatment of gut–brain disorders.

## Discussion

### M1 or M2: microglia are modified differently according to neuropathological contexts.

The engineering of microglia is bi-directional—one is promoting their polarisation towards an M1 (pro-inflammatory) phenotype, while the other is suppressing microglia hyperactivation to transform them into an M2 (anti-inflammatory) phenotype. Which direction to choose depends on the neuropathological context. In neurological diseases, where neuroinflammation is one of their causes, such as AD, in which misfolded protein aggregates induce excessive M1 microglia, more M2 microglia are warranted to suppress the adverse CNS immune response. However, in diseases, such as glioblastoma, where a stronger immune response is warranted to recognise and destroy cancer cells, more M1 microglia are required to enhance the CNS immune response by, for instance, the release of pro-inflammatory cytokines and recruitment of peripheral immune cells. Therefore, the engineering of microglia should be highly matched to the neuropathological contexts to which they are applied.

#### Beyond M1 and M2

Although most of the experimental evidence is built upon the canonical central tenets of the M1/M2 schema, it has been questioned whether M1/M2 polarisation does exist. First, instead of organising into a “spectrum” of activation states extending from M1 to M2, microglia can display “multidimensional” activation states in the shape of a 3D stellate polyhedron, with M1/M2 buried as baseline inside the polyhedron [137, 138]. Second, the coherence of M1/M2 marker expression is questionable. Although the usage of M1/M2 markers simplifies the determination of the expression profile and functional state of microglia, the results often fail to be replicated by unbiased expression profiling by scRNA-seq, due to the co-expression or absence of both polarisation markers [138–141]. Finally, the sequential microglial response to injury or infection—early M1 pro-inflammatory activities followed by M2 anti-inflammatory states beneficial for repair—is not consistently established and is context-dependent. The local environment and stimuli exposure together determine the outcomes [142]. For example, although M2 markers are generally anti-inflammatory and can alleviate neurodegeneration, ARG1 is a robust pro-neurodegeneration exception [119, 143]. Taking all these into consideration, we should be more careful when deploying M1/M2 polarisation as the outcome criterion of microglial engineering, as the markers chosen can be biased, not fully representing microglial transcriptomic profile, and might lead to unwanted functional states. Therefore, the contexts should be carefully considered to avoid misleading interpretations derived from M1/M2 polarisation.

Especially, emergent techniques such as scRNA-seq and spatial omics analyses have greatly challenged the classical view of M1 and M2. Increasingly resolved single-cell transcriptomics for microglia enable the transcriptome-based classification of microglia. This single-cell resolution leads to the discovery of far more microglial subtypes between the two extremes, M1 and M2, on the spectrum. Microglia demonstrate extensive spatial and temporal heterogeneity in different neuroanatomical destinations, as revealed by Masuda et al. [4]. From embryonic to postnatal stages of mice, spatiotemporal diversity of microglia subclasses decreases, by half, to only four new microglial subtypes, highly expressing microglial homeostatic genes Tmem119, Selplg and Slc2a5 [4]. Compared with the embryonic forebrain, IBA1^+^ microglia in the cortex dramatically eliminate the immunoreactivities of APOE and CTSB while unlocking CST3 and SPARC expression [4]. These expression profiles of microglia indicate their dynamic and differential roles in different spatial and temporal contexts, as comprehensively summarised in Masuda et al.’s review [5]. Elucidation of subtype-based functional heterogeneity has pros and cons for microglia re-engineering: on one hand, this increases targeting efficiency to specific spatiotemporal neuropathological contexts; on the other hand, however, this adds complexity and difficulty to controlling microglial states by genetic engineering or therapeutic modulation.

### Genetic targeting VS therapeutic modulation

Genetic targeting and therapeutic modulation are two mainstream methods of reprogramming microglia. Each has advantages and limitations. Genetic engineering of microglia has a more potent and direct effect, yet it is largely irreversible. Microglia in a “permanent” state, either M1 or M2 phenotype, are not beneficial for the long-term homeostasis of brain health. Henceforth, there should be a long-term activity monitoring of these genetically engineered microglia in the brain to prevent undesirable consequences. In contrast, therapeutic modulation of microglial activity is more reversible and tuneable, depending on the half-life and physiochemical properties of delivered therapeutics. However, off-target and side effects are more prominent in therapeutic modulation as therapeutics’ targeting efficiency on microglia is often unsatisfactory. Frequently, other non-microglia cells taking up these therapeutics will be affected functionally.

Therefore, the choice of re-engineering method should be carefully thought through and tailored to different neuropathological contexts, with various severeness degrees. For example, genetic engineering of microglia is preferred in late-stage AD than in early stage AD, as more potent and long-lasting therapeutic effects are warranted to attenuate or reverse severe AD symptoms. For milder AD, therapeutic effects offered by microglial modulation might be sufficient. Moreover, it is suggestible to combine two reprogramming strategies, when complications of different neurological diseases occur, to achieve an optimal clinical outcome. Therapeutic modulation can be coupled to modify the performance of genetically engineered microglia to bring out the maximal therapeutic benefits.

### No clinical evidence: why? Limitations and future directions

There is currently no clinical evidence for re-engineered microglia in treating neurological diseases in humans. This is probably due to (1) the re-engineering techniques are not mature and efficient enough, given that microglia fail to recapitulate in vitro states without CNS-derived cues, and microglia are often resistant to some forms of DNA manipulation; (2) the unignorable discrepancies between human and mouse microglia call for adjustments in engineering targets and delivery methods; (3) our understanding of microglia and their relationship with neurological diseases in humans is not complete and well-developed, making the results of applying engineered microglia full of uncertainties; (4) given the complexity and vulnerability of human CNS, treatment delivered to the brain should reach high levels of safety, which requires even more tests on animals, and thus the longer time required before clinical trials; (5) moreover, the targeting efficacy of microglia needs improvement to minimise off-target and side effects; and (6) the penetrability of therapeutics across BBB is always a primary concern. Certainly, there is still a long way to go for engineered microglia from bench to bedside. Henceforth, a deeper understanding of microglia’s role in CNS health and disease, and an improvement in engineered microglia’s targeting efficacy and BBB penetrability are warranted to enable them to treat neurological diseases in patients.

#### Future directions

One important step forward, as described in 2.5., is to expand in vitro experiments in human cell lines. Several platforms are currently available. One is for generating and manipulating human microglial states in different neuropathological contexts, while the others are for uncovering regulators of microglial disease states, leveraging the power of CRISPR technology. Utilising patient-derived microglia, incorporation of engineered microglia into 3D brain organoid models, and transplantation of engineered microglia into humanised animal models are future directions to advance our understanding of microglia’s role in different neuropathological diseases. Knowledge, e.g., key genes regulating microglial states, gained by screening using these platforms can provide more accurate engineering targets, generating re-programmed microglia that exert optimal therapeutic effects for neurological disease treatment.

Especially, incorporating engineered microglia into 3D brain organoid models facilitates the evaluation of their in vitro therapeutic efficacy in alleviating neuropathologies. Patient-derived brain organoids, at the interface of in vitro and in vivo, are a promising model that can recapitulate patients’ genetic architecture and in vivo neurophysiological pathological features. Compared with 2D stem cell lines, 3D brain organoids are advantageous in their self-organising ability and fuller recapitulation of the in vivo tissue architecture and physiological environments, enabling more multiplexed intercellular interactions [144]. Unlike astrocytes and oligodendrocytes, which develop from the neuroectoderm, microglia are derived from the embryonic mesoderm, the origin of the hematopoietic system. Therefore, the brain organoids often lack microglia and require external supplementation. There are several methods of integrating microglia: (1) vascularising the brain organoids could not only introduce microglia but also provide a constant supply of oxygen and nutrients, enabling better expansion of neural progenitors [145]; (2) coculturing hPSC-derived neural progenitor cells and primitive macrophage progenitors, which later differentiate into microglia [146]; and (3) directly seeding differentiated microglia into the brain organoids [147]. Besides these methods, however, Ormel et al. proposed that microglia can innately develop within cerebral organoids under proper induction, and these organoid-grown microglia demonstrated remarkable mimicry to those isolated from post-mortem human brain tissues [148]. It is suggestible to adopt Ormel et al.’s protocol to generate microglia-containing brain organoids that more fully recapitulate neuron–glia interactions in specific neuropathological contexts, enabling better evaluation of the modulatory effects of incorporated re-engineered microglia. Furthermore, as the in vitro incorporation is controllable temporally and spatially, extensive experimental testing enables the optimised and personalised delivery methods (doses, neuroanatomical location, injection frequency, etc.) in a clinical setting for different patients. In vivo transplantation of engineered human induced pluripotent stem cell-derived microglia with inhibitor-resistant CSF1R has been recently achieved to robustly replace endogenous microglia, indicating the promising translational values of engineered microglia transplantation [149].

Besides genetic targeting of microglia, modulation of microglial organelles can be a promising direction. One suggestible focus is mitochondria, the powerhouse of microglia. Mitophagy, the removal of damaged and superfluous mitochondria through autophagy, is crucial for maintaining proper microglial functions. Microglial mitophagy determines mitochondrial health, significantly influencing phagocytosis efficiency and controlling immune response as mitochondria coordinate the production of ROS and immune signalling molecules, key contributors to the neuroinflammation [150, 151]. Mitochondria released from fragmented microglia even activate astrocytes and propagate inflammatory neurodegeneration [152]. Microglial mitochondria dysfunction has thus been implicated in many neurological diseases. Henceforth, we suggest future therapeutic strategies target mitochondrial genetic deficits and restore their normal functions in microglia.

## Conclusion

Microglial engineering is an emerging strategy for treating neurological diseases. In this review, we have summarised the current development of genetic targeting and therapeutic manipulation of microglia to treat different neurological diseases, including neurodegenerative diseases, neuropsychiatric diseases, autoimmune diseases, and glioblastoma/glioma. Several delivery vehicles for therapeutics have been reviewed, ranging from nanoparticles to extracellular vesicles, and achieving microglial manipulation through interactions with other engineered cells, including stem cells and microbes. The pros and cons of each engineering method and factors impeding their clinical trials have also been discussed. This review aims to draw more attention to their potential and provide directions on which future research can work to propel the application of engineered microglia in treating various neurological disorders.

## Data Availability

Data sharing is not applicable to this article as no data sets were generated or analysed during the current study.

## Abbreviations

AD: Alzheimer’s disease
AGC: Anagecine
AZ: Azithromycin
BAG3: BCL-2-associated athanogene 3
BBB: Blood–brain barrier
Bryo-1: Bryostatin-1
CAR: Chimeric antigen receptors
CHL1: Cell adhesion molecule L1-like protein
CIONP: Coated iron oxide nanoparticles
CNS: Central nervous system
CPZ: Cuprizone
CSF-1: Colony-stimulating factor-1
CTLA-4: Cytotoxic T-lymphocyte-associated protein-4
DAM: Disease-associated microglia
EAE: Encephalomyelitis
ESdM: Microglia derived from embryonic stem cells
EV: Extracellular vesicles
FTD: Frontotemporal dementia
GA: Glatiramer acetate
GAM: Glioblastoma-associated microglia
GB: Glioblastoma
GLP-1: Glucagon-like peptide-1
GRN: Progranulin
GSC: Glioma stem cells
Ig: Immunoglobin
IL-34: Interleukin-34
LPS: Lipopolysaccharides
LTP: Long-term potentiation
MHC: Major histocompatibility complex
miRNA: MicroRNA
MITF: Melanocyte Inducing Transcription Factor
MMP3: Matrix metalloprotease 3
MS: Multiple sclerosis
ND: Neurodegenerative diseases
NK: Natural killer
NSC: Neural stem cell
NT-3: Neurotrophin-3
OGD: Oxygen–glucose deprivation
OL-HSPB8-EVs: Oligodendroglia-derived EVs enriched with heat shock protein B8
PEG: Polyethene glycol
PD: Parkinson's disease
PDGF-A: Platelet-derived growth factor A
pGlu3: Pyroglutamate-3
PGK: Phosphoglycerate kinase
PTX: Paclitaxel
rAAV2: Recombinant adeno-associated virus serotype 2
scRNAseq: Single-cell RNA sequencing
SDF-1: Stromal-derived cell factor 1
Sim: Simvastatin
TH: Tyrosine hydroxylase

## References

[1] Saijo K, Glass CK (2011). Microglial cell origin and phenotypes in health and disease. Nat Rev Immunol.

[2] Walton MR, Gibbons H, MacGibbon GA, Sirimanne E, Saura J, Gluckman PD, Dragunow M (2000). PU.1 expression in microglia. J Neuroimmunol.

[3] Rojo R, Raper A, Ozdemir DD, Lefevre L, Grabert K, Wollscheid-Lengeling E (2019). Deletion of a Csf1r enhancer selectively impacts CSF1R expression and development of tissue macrophage populations. Nat Commun.

[4] Masuda T, Sankowski R, Staszewski O, Bottcher C, Amann L, Scheiwe C (2019). Spatial and temporal heterogeneity of mouse and human microglia at single-cell resolution. Nature.

[5] Masuda T, Sankowski R, Staszewski O, Prinz M (2020). Microglia heterogeneity in the single-cell era. Cell Rep.

[6] Wang HS (2021). Microglia heterogeneity in Alzheimer’s disease: insights from single-cell technologies. Front Synaptic Neurosci..

[7] Franco-Bocanegra DK, Gourari Y, McAuley C, Chatelet DS, Johnston DA, Nicoll JAR, Boche D (2021). Microglial morphology in Alzheimer’s disease and after A beta immunotherapy. Sci Rep.

[8] Hayes GM, Woodroofe MN, Cuzner ML (1987). Microglia are the major cell type expressing MHC class-II in human white matter. J Neurol Sci.

[9] Lloyd AF, Davies CL, Holloway RK, Labrak Y, Ireland G, Carradori D (2019). Central nervous system regeneration is driven by microglia necroptosis and repopulation. Nat Neurosci.

[10] Galloway DA, Phillips AEM, Owen DRJ, Moore CS (2019). Phagocytosis in the brain: homeostasis and disease. Front Immunol.

[11] Magnus T, Chan A, Grauer O, Toyka KV, Gold R (2001). Microglial phagocytosis of apoptotic inflammatory T cells leads to down-regulation of microglial immune activation. J Immunol.

[12] Paolicelli RC, Bolasco G, Pagani F, Maggi L, Scianni M, Panzanelli P (2011). Synaptic pruning by microglia is necessary for normal brain development. Science.

[13] Schafer DP, Lehrman EK, Stevens B (2013). The, "quad-partite" synapse: Microglia-synapse interactions in the developing and mature CNS. Glia.

[14] Wang C, Yue HM, Hu ZC, Shen YW, Ma J, Li J (2020). Microglia mediate forgetting via complement-dependent synaptic elimination. Science.

[15] Wake H, Moorhouse AJ, Miyamoto A, Nabekura J (2013). Microglia: actively surveying and shaping neuronal circuit structure and function. Trends Neurosci.

[16] Liu XY, Quan N (2018). Microglia and CNS interleukin-1: beyond immunological concepts. Front Neurol.

[17] Recasens M, Almolda B, Perez-Clausell J, Campbell IL, Gonzalez B, Castellano B (2021). Chronic exposure to IL-6 induces a desensitized phenotype of the microglia. J Neuroinflamm.

[18] Kraft AD, McPherson CA, Harry GJ (2009). Heterogeneity of microglia and TNF signaling as determinants for neuronal death or survival. Neurotoxicology.

[19] Smith JA, Das A, Ray SK, Banik NL (2012). Role of pro-inflammatory cytokines released from microglia in neurodegenerative diseases. Brain Res Bull.

[20] Tejera D, Heneka MT, Garaschuk O, Verkhratsky A (2019). Microglia in neurodegenerative disorders. Microglia: methods and protocols. Methods in molecular biology.

[21] Doens D, Fernández PL (2014). Microglia receptors and their implications in the response to amyloid β for Alzheimer’s disease pathogenesis. J Neuroinflamm.

[22] Maphis N, Xu GX, Kokiko-Cochran ON, Jiang S, Cardona A, Ransohoff RM (2015). Reactive microglia drive tau pathology and contribute to the spreading of pathological tau in the brain. Brain.

[23] Chu FN, Shi MC, Zheng C, Shen DH, Zhu J, Zheng XY, Cui L (2018). The roles of macrophages and microglia in multiple sclerosis and experimental autoimmune encephalomyelitis. J Neuroimmunol.

[24] Calcia MA, Bonsall DR, Bloomfield PS, Selvaraj S, Barichello T, Howes OD (2016). Stress and neuroinflammation: a systematic review of the effects of stress on microglia and the implications for mental illness. Psychopharmacology.

[25] Prinz M, Priller J (2014). Microglia and brain macrophages in the molecular age: from origin to neuropsychiatric disease. Nat Rev Neurosci.

[26] Gutmann DH, Kettenmann H (2019). Microglia/brain macrophages as central drivers of brain tumor pathobiology. Neuron.

[27] Maas SLN, Abels ER, Van De Haar LL, Zhang X, Morsett L, Sil S (2020). Glioblastoma hijacks microglial gene expression to support tumor growth. J Neuroinflamm.

[28] Geribaldi-Doldan N, Fernandez-Ponce C, Quiroz RN, Sanchez-Gomar I, Escorcia LG, Velasquez EP, Quiroz EN (2021). The role of microglia in glioblastoma. Front Oncol.

[29] Matias D, Balca-Silva J, da Graca GC, Wanjiru CM, Macharia LW, Nascimento CP (2018). Microglia/astrocytes-glioblastoma crosstalk: crucial molecular mechanisms and microenvironmental factors. Front Cell Neurosci.

[30] Garofalo S, Porzia A, Mainiero F, Di Angelantonio S, Cortese B, Basilico B (2017). Environmental stimuli shape microglial plasticity in glioma. Elife.

[31] Mormino A, Bernardini G, Cocozza G, Corbi N, Passananti C, Santoni A (2021). Enriched environment cues suggest a new strategy to counteract glioma: engineered rAAV2-IL-15 microglia modulate the tumor microenvironment. Front Immunol.

[32] Su W, Kang J, Sopher B, Gillespie J, Aloi MS, Odom GL (2016). Recombinant adeno-associated viral (rAAV) vectors mediate efficient gene transduction in cultured neonatal and adult microglia. J Neurochem.

[33] Zhang HW, Yang B, Mu X, Ahmed SS, Su Q, He R (2011). Several rAAV vectors efficiently cross the blood-brain barrier and transduce neurons and astrocytes in the neonatal mouse central nervous system. Mol Ther.

[34] Rosario AM, Cruz PE, Ceballos-Diaz C, Strickland MR, Siemienski Z, Pardo M (2016). Microglia-specific targeting by novel capsid- modified AAV6 vectors. Mol Ther Methods Clin Dev..

[35] Ye Z, Ai X, Yang K, Yang Z, Fei F, Liao X (2023). Targeting microglial metabolic rewiring synergizes with immune-checkpoint blockade therapy for glioblastoma. Cancer Discov.

[36] Guo L, Zhang XC, Wei RX, Li GJ, Sun BZ, Zhang HB (2020). Engineering microglia as intraoperative optical imaging agent vehicles potentially for fluorescence-guided surgery in gliomas. Biomater Sci.

[37] Du YT, Yang ZZ, Sun Q, Lin M, Wang RD, Peng YW (2021). Engineered microglia potentiate the action of drugs against glioma through extracellular vesicles and tunneling nanotubes. Adv Healthc Mater..

[38] Bhattacherjee A, Daskhan GC, Bains A, Watson AES, Eskandari-Sedighi G, St Laurent CD (2021). Increasing phagocytosis of micoglia by targeting CD33 with liposomes displaying glycan ligands. J Control Release.

[39] Vermeer CJC, Hiensch AE, Cleenewerk L, May AM, Eijkelkamp N (2021). Neuro-immune interactions in paclitaxel-induced peripheral neuropathy. Acta Oncol.

[40] Staff NP, Fehrenbacher JC, Caillaud M, Damaj MI, Segal RA, Rieger S (2020). Pathogenesis of paclitaxel-induced peripheral neuropathy: a current review of in vitro and in vivo findings using rodent and human model systems. Exp Neurol.

[41] Tang M, Zhao S, Liu JX, Liu X, Guo YX, Wang GY, Wang XL (2022). Paclitaxel induces cognitive impairment via necroptosis, decreased synaptic plasticity and M1 polarisation of microglia. Pharm Biol.

[42] Gao XH, Li S, Ding F, Liu XL, Wu YJ, Li J (2021). A virus-mimicking nucleic acid nanogel reprograms microglia and macrophages for glioblastoma therapy. Adv Mater.

[43] Abels ER, Maas SLN, Nieland L, Wei ZY, Cheah PS, Tai E (2019). Glioblastoma-associated microglia reprogramming is mediated by functional transfer of extracellular miR-21. Cell Rep.

[44] Guo YW, Hong WM, Wang XM, Zhang PY, Korner H, Tu JJ, Wei W (2019). MicroRNAs in microglia: how do microRNAs affect activation, inflammation, polarization of microglia and mediate the interaction between microglia and glioma?. Front Mol Neurosci.

[45] Louw AM, Kolar MK, Novikova LN, Kingham PJ, Wiberg M, Kjems J, Novikov LN (2016). Chitosan polyplex mediated delivery of miRNA-124 reduces activation of microglial cells in vitro and in rat models of spinal cord injury. Nanomedicine.

[46] Banelli B, Forlani A, Allemanni G, Morabito A, Pistillo MP, Romani M (2017). MicroRNA in glioblastoma: an overview. Int J Genomics..

[47] Hickman S, Izzy S, Sen P, Morsett L, El Khoury J (2018). Microglia in neurodegeneration. Nat Neurosci.

[48] Shao FJ, Wang XY, Wu HJ, Wu Q, Zhang JM (2022). Microglia and neuroinflammation: crucial pathological mechanisms in traumatic brain injury-induced neurodegeneration. Front Aging Neurosci..

[49] Fiebich BL, Batista CRA, Saliba SW, Yousif NM, de Oliveira ACP (2018). Role of microglia TLRs in neurodegeneration. Front Cell Neurosci.

[50] Plasschaert RN, DeAndrade MP, Hull F, Nguyen Q, Peterson T, Yan A (2022). High-throughput analysis of hematopoietic stem cell engraftment after intravenous and intracerebroventricular dosing. Mol Ther.

[51] Rocha EM, Smith GA, Park E, Cao HM, Brown E, Hayes MA (2015). Glucocerebrosidase gene therapy prevents alpha-synucleinopathy of midbrain dopamine neurons. Neurobiol Dis.

[52] Arrant AE, Onyilo VC, Unger DE, Roberson ED (2018). Progranulin gene therapy improves lysosomal dysfunction and microglial pathology associated with frontotemporal dementia and neuronal ceroid lipofuscinosis. J Neurosci.

[53] Minami SS, Min SW, Krabbe G, Wang C, Zhou YG, Asgarov R (2014). Progranulin protects against amyloid beta deposition and toxicity in Alzheimer’s disease mouse models. Nat Med.

[54] Hu XY, Das B, Hou HL, He WX, Yan RQ (2018). BACE1 deletion in the adult mouse reverses preformed amyloid deposition and improves cognitive functions. J Exp Med.

[55] Vassar R, Kuhn PH, Haass C, Kennedy ME, Rajendran L, Wong PC, Lichtenthaler SF (2014). Function, therapeutic potential and cell biology of BACE proteases: current status and future prospects. J Neurochem.

[56] Kim W, Ma L, Lomoio S, Willen R, Lombardo S, Dong JH (2018). BACE1 elevation engendered by GGA3 deletion increases beta-amyloid pathology in association with APP elevation and decreased CHL1 processing in 5XFAD mice. Mol Neurodegener.

[57] Lombardo S, Chiacchiaretta M, Tarr A, Kim W, Cao TY, Sigal G (2019). BACE1 partial deletion induces synaptic plasticity deficit in adult mice. Sci Rep.

[58] Wang HX, He Y, Sun ZL, Ren SY, Liu MX, Wang G, Yang J (2022). Microglia in depression: an overview of microglia in the pathogenesis and treatment of depression. J Neuroinflamm.

[59] Cosma NC, Usekes B, Otto LR, Gerike S, Heuser I, Regen F, Hellmann-Regen J (2021). M1/M2 polarization in major depressive disorder: Disentangling state from trait effects in an individualized cell-culture-based approach. Brain Behav Immun.

[60] Zhang LJ, Zhang JQ, You ZL (2018). Switching of the microglial activation phenotype is a possible treatment for depression disorder. Front Cell Neurosci.

[61] Liu Y, Hu P, Zheng ZH, Zhong D, Xie WC, Tang ZB (2022). Photoresponsive vaccine-like CAR-M system with high-efficiency central immune regulation for inflammation-related depression. Adv Mater.

[62] Wang SH, Yang YQ, Ma PW, Zha Y, Zhang J, Lei AH, Li N (2022). CAR-macrophage: an extensive immune enhancer to fight cancer. EBioMedicine.

[63] Klichinsky M, Ruella M, Shestova O, Lu XM, Best A, Zeeman M (2020). Human chimeric antigen receptor macrophages for cancer immunotherapy. Nat Biotechnol.

[64] Gatto L, Di Nunno V, Franceschi E, Brandes AA (2021). Chimeric antigen receptor macrophage for glioblastoma immunotherapy: the way forward. Immunotherapy.

[65] Chen C, Jing W, Chen Y, Wang G, Abdalla M, Gao L (2022). Intracavity generation of glioma stem cell–specific CAR macrophages primes locoregional immunity for postoperative glioblastoma therapy. Sci Transl Med.

[66] Rossari F, Birocchi F, Cusimano M, Ranghetti A, Orofino G, Sergi LS (2021). Interferon-alpha gene delivery by tumor-associated macrophages improves function and prevents exhaustion of B7-H3-redirected CAR T cells in glioblastoma. Hum Gene Therapy..

[67] Jurga AM, Paleczna M, Kuter KZ (2020). Overview of general and discriminating markers of differential microglia phenotypes. Front Cell Neurosci.

[68] Sevenich L (2018). Brain-resident microglia and blood-borne macrophages orchestrate central nervous system inflammation in neurodegenerative disorders and brain cancer. Front Immunol.

[69] Shemer A, Grozovski J, Tay TL, Tao J, Volaski A, Süß P (2018). Engrafted parenchymal brain macrophages differ from microglia in transcriptome, chromatin landscape and response to challenge. Nat Commun.

[70] Bhagavati S (2021). Autoimmune disorders of the nervous system: pathophysiology, clinical features, and therapy. Front Neurol.

[71] Luo C, Jian CD, Liao YH, Huang Q, Wu YJ, Liu XX (2017). The role of microglia in multiple sclerosis. Neuropsych Dis Treat.

[72] Tzeng SF, Huang HY (2003). Downregulation of inducible nitric oxide synthetase by neurotrophin-3 in microglia. J Cell Biochem.

[73] Elkabes S, DiCiccoBloom EM, Black IB (1996). Brain microglia macrophages express neurotrophins that selectively regulate microglial proliferation and function. J Neurosci.

[74] Neumann H, Misgeld T, Matsumuro K, Wekerle H (1998). Neurotrophins inhibit major histocompatibility class II inducibility of microglia: involvement of the p75 neurotrophin receptor. Proc Natl Acad Sci USA.

[75] Beutner C, Lepperhof V, Dann A, Linnartz-Gerlach B, Litwak S, Napoli I (2013). Engineered stem cell-derived microglia as therapeutic vehicle for experimental autoimmune encephalomyelitis. Gene Ther.

[76] Aharoni R, Eilam R, Domev H, Labunskay G, Sela M, Arnon R (2005). The immunomodulator glatiramer acetate augments the expression of neurotrophic factors in brains of experimental autoimmune encephalomyelitis mice. Proc Natl Acad Sci USA.

[77] Casella G, Colombo F, Finardi A, Descamps H, Ill-Raga G, Spinelli A (2018). Extracellular vesicles containing IL-4 modulate neuroinflammation in a mouse model of multiple sclerosis. Mol Ther.

[78] Dolan MJ, Therrien M, Jereb S, Kamath T, Gazestani V, Atkeson T (2023). Exposure of iPSC-derived human microglia to brain substrates enables the generation and manipulation of diverse transcriptional states in vitro. Nat Immunol.

[79] Dräger NM, Sattler SM, Huang CT-L, Teter OM, Leng K, Hashemi SH (2022). A CRISPRi/a platform in human iPSC-derived microglia uncovers regulators of disease states. Nat Neurosci.

[80] Chang JC-Y, Wang C-Y, Lin S (2023). Interrogation of human microglial phagocytosis by CRISPR genome editing. Front Immunol.

[81] Yu ZW, Sun DY, Feng JF, Tan WX, Fang X, Zhao M (2015). MSX3 switches microglia polarization and protects from inflammation-induced demyelination. J Neurosci.

[82] Grimaldi A, D'Alessandro G, Golia MT, Grossinger EM, Di Angelantonio S, Ragozzino D (2016). KCa3.1 inhibition switches the phenotype of glioma-infiltrating microglia/macrophages. Cell Death Dis.

[83] Tóbon-Velasco JC, Cuevas E, Torres-Ramos MA (2014). Receptor for AGEs (RAGE) as mediator of NF-kB pathway activation in neuroinflammation and oxidative stress. CNS Neurol Disord Drug Targets.

[84] Deane RJ (2012). Is RAGE still a therapeutic target for Alzheimer’s disease?. Future Med Chem.

[85] Gasparotto J, Ribeiro CT, Bortolin RC, Somensi N, Rabelo TK, Kunzler A (2017). Targeted inhibition of RAGE in substantia nigra of rats blocks 6-OHDA–induced dopaminergic denervation. Sci Rep-Uk.

[86] Teismann P, Sathe K, Bierhaus A, Leng L, Martin H, Bucala R (2012). Receptor for advanced glycation endproducts (RAGE) deficiency protects against MPTP toxicity. Neurobiol Aging.

[87] Santos G, Barateiro A, Brites D, Fernandes A (2020). S100B impairs oligodendrogenesis and myelin repair following demyelination through RAGE engagement. Front Cell Neurosci.

[88] Leclerc E, Sturchler E, Vetter SW (2010). The S100B/RAGE axis in Alzheimer's disease. Cardiovasc Psychiatry Neurol.

[89] Xie X, Luo X, Liu N, Li X, Lou F, Zheng Y, Ren Y (2017). Monocytes, microglia, and CD200-CD200R1 signaling are essential in the transmission of inflammation from the periphery to the central nervous system. J Neurochem.

[90] Rabaneda-Lombarte N, Serratosa J, Bové J, Vila M, Saura J, Solà C (2021). The CD200R1 microglial inhibitory receptor as a therapeutic target in the MPTP model of Parkinson's disease. J Neuroinflammation.

[91] Rabaneda-Lombarte N, Vidal-Taboada JM, Valente T, Ezquerra M, Fernández-Santiago R, Martí MJ (2022). Altered expression of the immunoregulatory ligand-receptor pair CD200-CD200R1 in the brain of Parkinson’s disease patients. Npj Parkinsons Dis..

[92] Liu Y, Bando Y, Vargas-Lowy D, Elyaman W, Khoury SJ, Huang T (2010). CD200R1 agonist attenuates mechanisms of chronic disease in a murine model of multiple sclerosis. J Neurosci.

[93] Wilkinson K, El Khoury J (2012). Microglial scavenger receptors and their roles in the pathogenesis of Alzheimer’s disease. Int J Alzheimers Dis.

[94] Ulland TK, Colonna M (2018). TREM2: a key player in microglial biology and Alzheimer disease. Nat Rev Neurol.

[95] Peng Q, Malhotra S, Humphrey MB (2010). Association of TREM2-DAP12 with DAP10 is required for the regulation of PI3K in macrophages (98.18). J Immunol.

[96] Sayed FA, Kodama L, Fan L, Carling GK, Udeochu JC, Le D (2021). AD-linked R47H-TREM2 mutation induces disease-enhancing microglial states via AKT hyperactivation. Sci Transl Med..

[97] Huang Y, Happonen KE, Burrola PG, O’Connor C, Hah N, Huang L (2021). Microglia use TAM receptors to detect and engulf amyloid β plaques. Nat Immunol.

[98] Butler CA, Popescu AS, Kitchener EJA, Allendorf DH, Puigdellívol M, Brown GC (2021). Microglial phagocytosis of neurons in neurodegeneration, and its regulation. J Neurochem.

[99] Haynes SE, Hollopeter G, Yang G, Kurpius D, Dailey ME, Gan WB, Julius D (2006). The P2Y12 receptor regulates microglial activation by extracellular nucleotides. Nat Neurosci.

[100] Lauro C, Catalano M, Trettel F, Mainiero F, Ciotti MT, Eusebi F, Limatola C (2006). The chemokine CX3CL1 reduces migration and increases adhesion of neurons with mechanisms dependent on the beta1 integrin subunit. J Immunol.

[101] Klaus C, Liao H, Allendorf DH, Brown GC, Neumann H (2021). Sialylation acts as a checkpoint for innate immune responses in the central nervous system. Glia.

[102] Lehrman EK, Wilton DK, Litvina EY, Welsh CA, Chang ST, Frouin A (2018). CD47 protects synapses from excess microglia-mediated pruning during development. Neuron.

[103] Deane R, Sagare A, Hamm K, Parisi M, LaRue B, Guo H (2005). IgG-assisted age-dependent clearance of Alzheimer's amyloid beta peptide by the blood-brain barrier neonatal Fc receptor. J Neurosci.

[104] Kim C, Ho DH, Suk JE, You S, Michael S, Kang J (2013). Neuron-released oligomeric α-synuclein is an endogenous agonist of TLR2 for paracrine activation of microglia. Nat Commun.

[105] Choi I, Zhang YX, Seegobin SP, Pruvost M, Wang Q, Purtell K (2020). Microglia clear neuron-released a-synuclein via selective autophagy and prevent neurodegeneration. Nat Commun.

[106] Brelstaff JH, Mason M, Katsinelos T, McEwan WA, Ghetti B, Tolkovsky AM, Spillantini MG (2021). Microglia become hypofunctional and release metalloproteases and tau seeds when phagocytosing live neurons with P301S tau aggregates. Sci Adv.

[107] Chiu T-L, Wang M-J, Su C-C (2012). The treatment of glioblastoma multiforme through activation of microglia and TRAIL induced by rAAV2-mediated IL-12 in a syngeneic rat model. J Biomed Sci.

[108] McAlpine CS, Park J, Griciuc A, Kim E, Choi SH, Iwamoto Y (2021). Astrocytic interleukin-3 programs microglia and limits Alzheimer’s disease. Nature.

[109] Baik SH, Kang S, Lee W, Choi H, Chung S, Kim J-I, Mook-Jung I (2019). A breakdown in metabolic reprogramming causes microglia dysfunction in Alzheimer’s disease. Cell Metab.

[110] Crehan H, Liu B, Kleinschmidt M, Rahfeld J-U, Le KX, Caldarone BJ (2020). Effector function of anti-pyroglutamate-3 Aβ antibodies affects cognitive benefit, glial activation and amyloid clearance in Alzheimer’s-like mice. Alzheimers Res Ther.

[111] Reifschneider A, Robinson S, van Lengerich B, Gnörich J, Logan T, Heindl S (2022). Loss of TREM2 rescues hyperactivation of microglia, but not lysosomal deficits and neurotoxicity in models of progranulin deficiency. Embo J.

[112] Butovsky O, Jedrychowski MP, Cialic R, Krasemann S, Murugaiyan G, Fanek Z (2015). Targeting miR-155 restores abnormal microglia and attenuates disease in SOD1 mice. Ann Neurol.

[113] Sun D, Yu Z, Fang X, Liu M, Pu Y, Shao Q (2017). LncRNA GAS5 inhibits microglial M2 polarization and exacerbates demyelination. EMBO Rep.

[114] Wang G, Shi Y, Jiang X, Leak RK, Hu X, Wu Y (2015). HDAC inhibition prevents white matter injury by modulating microglia/macrophage polarization through the GSK3β/PTEN/Akt axis. Proc Natl Acad Sci USA.

[115] Fan Y, Li Y, Yang Y, Lin K, Lin Q, Luo S (2022). Chlorogenic acid prevents microglia-induced neuronal apoptosis and oxidative stress under hypoxia-ischemia environment by regulating the MIR497HG/miR-29b-3p/SIRT1 axis. Dis Markers.

[116] Miles LA, Hermans SJ, Crespi GAN, Gooi JH, Doughty L, Nero TL (2019). Small molecule binding to alzheimer risk factor CD33 promotes Aβ phagocytosis. iScience..

[117] Zhao NX, Francis NL, Calvelli HR, Moghe PV (2020). Microglia-targeting nanotherapeutics for neurodegenerative diseases. Apl Bioeng..

[118] Amantea D, Certo M, Petrelli F, Bagetta G (2016). Neuroprotective properties of a macrolide antibiotic in a mouse model of middle cerebral artery occlusion: characterization of the immunomodulatory effects and validation of the efficacy of intravenous administration. Assay Drug Dev Technol.

[119] Pesce JT, Ramalingam TR, Mentink-Kane MM, Wilson MS, El Kasmi KC, Smith AM (2009). Arginase-1-expressing macrophages suppress Th2 cytokine-driven inflammation and fibrosis. PLoS Pathog.

[120] Joseph A, Liao R, Zhang MY, Helmbrecht H, McKenna M, Filteau JR, Nance E (2020). Nanoparticle-microglial interaction in the ischemic brain is modulated by injury duration and treatment. Bioeng Transl Med..

[121] Yang CC, Gong SL, Chen XP, Wang MY, Zhang L, Zhang L, Hu CY (2021). Analgecine regulates microglia polarization in ischemic stroke by inhibiting NF-rB through the TLR4 MyD88 pathway. Int Immunopharmacol.

[122] Zhou JM, Gu SS, Mei WH, Zhou J, Wang ZZ, Xiao W (2016). Ginkgolides and bilobalide protect BV2 microglia cells against OGD/reoxygenation injury by inhibiting TLR2/4 signaling pathways. Cell Stress Chaperones.

[123] Manickavasagam D, Novak K, Oyewumi MO (2018). Therapeutic delivery of simvastatin loaded in PLA-PEG polymersomes resulted in amplification of anti-inflammatory effects in activated microglia. Aaps J.

[124] Emmerich K, White DT, Kambhampati SP, Casado GL, Fu T-M, Chunawala Z (2023). Nanoparticle-based targeting of microglia improves the neural regeneration enhancing effects of immunosuppression in the zebrafish retina. Commun Biol.

[125] Wu WC, Tian J, Xiao D, Guo YX, Xiao Y, Wu XY (2022). Engineered extracellular vesicles encapsulated Bryostatin-1 as therapy for neuroinflammation. Nanoscale.

[126] Li X, Tian J, Zhang Y (2019). Targeting CNS extracellular vesicles enhanced bryostatin-1 therapeutic effect on experimental autoimmune encephalomyelitis. Eur J Immunol.

[127] Van den Broek B, Wuyts C, Sisto A, Pintelon I, Timmermans JP, Somers V (2022). Oligodendroglia-derived extracellular vesicles activate autophagy via LC3B/BAG3 to protect against oxidative stress with an enhanced effect for HSPB8 enriched vesicles. Cell Commun Signal.

[128] Le Blon D, Guglielmetti C, Hoornaert C, Quarta A, Daans J, Dooley D (2016). Intracerebral transplantation of interleukin 13-producing mesenchymal stem cells limits microgliosis, oligodendrocyte loss and demyelination in the cuprizone mouse model. J Neuroinflamm.

[129] Abdel-Haq R, Schlachetzki JCM, Glass CK, Mazmanian SK (2019). Microbiome-microglia connections via the gut-brain axis. J Exp Med.

[130] Wang YL, Wang ZY, Wang Y, Li F, Jia JY, Song XW (2018). The gut-microglia connection: implications for central nervous system diseases. Front Immunol.

[131] Houser MC, Tansey MG (2017). The gut-brain axis: is intestinal inflammation a silent driver of Parkinson's disease pathogenesis?. Npj Parkinsons Dis..

[132] Garcia-Dominguez I, Vesela K, Garcia-Revilla J, Carrillo-Jimenez A, Roca-Ceballos MA, Santiago M (2018). Peripheral inflammation enhances microglia response and nigral dopaminergic cell death in an in vivo MPTP model of Parkinson’s disease. Front Cell Neurosci.

[133] Di Meo F, Margarucci S, Galderisi U, Crispi S, Peluso G (2019). Curcumin, gut microbiota, and neuroprotection. Nutrients.

[134] Petrella C, Strimpakos G, Torcinaro A, Middei S, Ricci V, Gargari G (2021). Proneurogenic and neuroprotective effect of a multi strain probiotic mixture in a mouse model of acute inflammation: involvement of the gut-brain axis. Pharmacol Res.

[135] Wu H, Wei J, Zhao XM, Liu Y, Chen ZH, Wei KH (2022). Neuroprotective effects of an engineered * Escherichia *
* coli * Nissle 1917 on Parkinson’s disease in mice by delivering GLP-1 and modulating gut microbiota. Bioeng Transl Med.

[136] Sanchez-Guajardo V, Tentillier N, Romero-Ramos M (2015). The relation between alpha-synuclein and microglia in parkinson's disease: recent developments. Neuroscience.

[137] Xue J, Schmidt SV, Sander J, Draffehn A, Krebs W, Quester I (2014). Transcriptome-based network analysis reveals a spectrum model of human macrophage activation. Immunity.

[138] Ransohoff RM (2016). A polarizing question: do M1 and M2 microglia exist?. Nat Neurosci.

[139] Morganti JM, Riparip L-K, Rosi S (2016). Call off the dog (ma): M1/M2 polarization is concurrent following traumatic brain injury. PLoS ONE.

[140] Kim CC, Nakamura MC, Hsieh CL (2016). Brain trauma elicits non-canonical macrophage activation states. J Neuroinflammation.

[141] Zhang Y, Chen K, Sloan SA, Bennett ML, Scholze AR, O'Keeffe S (2014). An RNA-sequencing transcriptome and splicing database of glia, neurons, and vascular cells of the cerebral cortex. J Neurosci.

[142] Gosselin D, Link VM, Romanoski CE, Fonseca GJ, Eichenfield DZ, Spann NJ (2014). Environment drives selection and function of enhancers controlling tissue-specific macrophage identities. Cell.

[143] Kan MJ, Lee JE, Wilson JG, Everhart AL, Brown CM, Hoofnagle AN (2015). Arginine deprivation and immune suppression in a mouse model of Alzheimer's disease. J Neurosci.

[144] Chiaradia I, Lancaster MA (2020). Brain organoids for the study of human neurobiology at the interface of in vitro and in vivo. Nat Neurosci.

[145] Cakir B, Park IH (2022). Getting the right cells. Elife.

[146] Xu RJ, Boreland AJ, Li XX, Erickson C, Jin MM, Atkins C (2021). Developing human pluripotent stem cell-based cerebral organoids with a controllable microglia ratio for modeling brain development and pathology. Stem Cell Rep.

[147] Sabate-Soler S, Nickels SL, Saraiva C, Berger E, Dubonyte U, Barmpa K (2022). Microglia integration into human midbrain organoids leads to increased neuronal maturation and functionality. Glia.

[148] Ormel PR, de Sa RV, van Bodegraven EJ, Karst H, Harschnitz O, Sneeboer MAM (2018). Microglia innately develop within cerebral organoids. Nat Commun.

[149] Chadarevian JP, Lombroso SI, Peet GC, Hasselmann J, Tu C, Marzan DE (2022). Engineering an inhibitor-resistant human CSF1R variant for microglia replacement. J Exp Med.

[150] Agrawal I, Jha S (2020). Mitochondrial dysfunction and Alzheimer's disease: role of microglia. Front Aging Neurosci..

[151] Fairley LH, Wong JH, Barron AM (2021). Mitochondrial regulation of microglial immunometabolism in Alzheimers disease. Front Immunol.

[152] Joshi AU, Minhas PS, Liddelow SA, Haileselassie B, Andreasson KI, Dorn GW, Mochly-Rosen D (2019). Fragmented mitochondria released from microglia trigger A1 astrocytic response and propagate inflammatory neurodegeneration. Nat Neurosci.

